# Effects of a computerised diagnostic decision support tool on diagnostic quality in emergency departments: study protocol of the DDx-BRO multicentre cluster randomised cross-over trial

**DOI:** 10.1136/bmjopen-2023-072649

**Published:** 2023-03-28

**Authors:** Thimo Marcin, Stefanie C Hautz, Hardeep Singh, Laura Zwaan, David Schwappach, Gert Krummrey, Stefan K Schauber, Mathieu Nendaz, Aristomenis Konstantinos Exadaktylos, Martin Müller, Cornelia Lambrigger, Thomas C Sauter, Gregor Lindner, Simon Bosbach, Ines Griesshammer, Wolf E Hautz

**Affiliations:** 1 Department of Emergency Medicine, Inselspital, Bern University Hospital, University of Bern, Bern, Switzerland; 2 Center for Innovations in Quality, Effectiveness and Safety (IQuESt), Michael E DeBakey VA Medical Center, Houston, Texas, USA; 3 Department of Medicine, Baylor College of Medicine, Houston, Texas, USA; 4 Institute of Medical Education Research Rotterdam (iMERR), Erasmus Medical Center, Rotterdam, The Netherlands; 5 Institute of Social and Preventive Medicine (ISPM), University of Bern, Bern, Switzerland; 6 Bern University of Applied Sciences, Biel, Switzerland; 7 Center for Educational Measurement and Faculty of Medicine, University of Oslo, Oslo, Norway; 8 Department of Medicine, University of Geneva, Geneve, Switzerland; 9 Department of Internal and Emergency Medicine, Burgerspital Solothurn, Solothurn, Switzerland; 10 Spital Tiefenau, Insel Gruppe AG, Bern, Switzerland; 11 Spital Muensingen, Insel Gruppe AG, Bern, Switzerland

**Keywords:** accident & emergency medicine, health informatics, information management, decision making, quality in health care, internal medicine

## Abstract

**Introduction:**

Computerised diagnostic decision support systems (CDDS) suggesting differential diagnoses to physicians aim to improve clinical reasoning and diagnostic quality. However, controlled clinical trials investigating their effectiveness and safety are absent and the consequences of its use in clinical practice are unknown. We aim to investigate the effect of CDDS use in the emergency department (ED) on diagnostic quality, workflow, resource consumption and patient outcomes.

**Methods and analysis:**

This is a multicentre, outcome assessor and patient-blinded, cluster-randomised, multiperiod crossover superiority trial. A validated differential diagnosis generator will be implemented in four EDs and randomly allocated to a sequence of six alternating intervention and control periods. During intervention periods, the treating ED physician will be asked to consult the CDDS at least once during diagnostic workup. During control periods, physicians will not have access to the CDDS and diagnostic workup will follow usual clinical care. Key inclusion criteria will be patients’ presentation to the ED with either fever, abdominal pain, syncope or a non-specific complaint as chief complaint. The primary outcome is a binary diagnostic quality risk score composed of presence of an unscheduled medical care after discharge, change in diagnosis or death during time of follow-up or an unexpected upscale in care within 24 hours after hospital admission. Time of follow-up is 14 days. At least 1184 patients will be included. Secondary outcomes include length of hospital stay, diagnostics and data regarding CDDS usage, physicians’ confidence calibration and diagnostic workflow. Statistical analysis will use general linear mixed modelling methods.

**Ethics and dissemination:**

Approved by the cantonal ethics committee of canton Berne (2022-D0002) and Swissmedic, the Swiss national regulatory authority on medical devices. Study results will be disseminated through peer-reviewed journals, open repositories and the network of investigators and the expert and patients advisory board.

**Trial registration number:**

NCT05346523.

**STRENGTHS AND LIMITATIONS OF THIS STUDY**

- Study addresses diagnostic error in emergency medicine, a significant patient safety topic which is under-researched.
- First prospective randomised clinical trial on the effect of computerised diagnostic decision support programmes in a real-world setting.
- Patient relevant outcomes, including mortality and unscheduled revisits.
- Multicentre cluster randomised crossover trial with blinded outcome assessment.
- Non-random selection of participating emergency rooms.

## Introduction

### Diagnostic error in emergency medicine

Getting the right diagnosis in healthcare is key to provide an explanation of a patient’s health problem and informs subsequent healthcare and treatment.^1^ ^2 3^ We have previously found clinically significant diagnostic discrepancies between emergency department (ED) and hospital discharge diagnosis in 12.3% of the patients, which were associated with longer hospital stay and increased mortality.^4^ The causes for diagnostic error can be diverse, but one major cause is human error.^1 3 5^ This includes the consideration of incomplete patient histories, failure to consider alternative diagnoses, lack of knowledge and lack of recognition of clinical findings by physicians.^5–9^ An accurate diagnosis is the basis for all treatment and care and hence, improving the diagnostic process and accuracy is key to improving patient safety and outcome.

### Clinical decision support systems (CDS)

Within the last decades, digitalisation in healthcare has led to a rapid evolution of various CDS supposed to augment clinicians in their complex decision-making processes.^10^ A recent systematic literature review on electronic health record (EHR)-integrated generic CDS in the ED found positive effects on various outcomes in 83% of the included articles.^11^ However, they included various CDS types with heterogeneous targets and measured outcomes and studies of mixed quality. CDS that were built to support clinicians specifically in the process of diagnosis (computerised diagnostic decision support system (CDDS)) have not shown such promising results as other types of CDS so far, mostly due to negative physician perception and biases, poor accuracy or poor system integration.^10^

### Differential diagnosis

A key role in the diagnostic process has been attributed to differential diagnoses (DDx), as they guide physicians in considering or excluding possible diagnoses in the ongoing diagnostic process.^12^ Research on diagnostic decision-making found that in the absence of a correct diagnostic hypothesis, physicians tried to explain evidence away that did not fit their diagnosis and that misdiagnosis occurred most when the correct diagnosis was not even considered.^13 14^ Broadening the differentials has been consistently recognised as an important measure to avoid diagnostic errors.^15 16^ But given the vast amount of diseases and clinical manifestations that ED physicians are confronted with, they cannot be expected to think of all possible DDx. It is likely that they either do not recall or know all of the potential DDx fitting the clinical presentation and symptoms of any given patient.

### DDx generators

Reminding physicians of potential DDx is the aim of DDx generators. DDx generators are built to provide physicians with a list of DDx based on clinical data input, such as patient characteristics, symptoms, findings and other factors, and were first developed in the 1970s. Since then, DDx generators have evolved in the last decades as computational methods have advanced. Nowadays, DDx generators are capable of matching input data to large electronic databases of diagnoses using varying computational methods such as Bayesian probabilities or text mining techniques,^17^ and to subsequently retrieve the correct diagnosis with an acceptable accuracy. Likewise, integration in EHR systems has improved continuously and enhanced usability in clinical practice.^10^

### Accuracy of DDx generators

A systematic review and meta-analysis from 2016 investigating the efficacy and utility of DDx generators found a pooled accuracy of computer suggested diagnoses of 0.7, meaning that the correct diagnosis was among the suggested DDx in 70% of the cases. However, only small improvements were seen in before and after studies, where clinicians had the opportunity to revisit their diagnoses following a DDx generator consultation.^17^ However, most of the included studies were considered to be at high risk for selection, funding and publication bias and the findings of some older included studies may not be applicable to the new generation of DDx generators, given their improved accuracy and usability. Most importantly, DDx generators were applied retrospectively across the majority of the studies, reducing the external validity of the results.

### Effectiveness of DDx generators and knowledge gaps

To the best of our knowledge, studies investigating the effectiveness and safety of DDx generators in ‘real-world’ clinical setting are absent. Therefore, it is unknown to what extent the use of these CDDS actually improves the quality of medical diagnoses and consecutive health outcomes of the individual patient. In addition, research indicates that collaboration among clinicians is frequent and substantially improves diagnostic accuracy.^1 18 19^ CDDS may lower ED physician’s perception of whether they need advice from other team members, and thus reduce the likelihood of collaboration. The effect of a DDx generator on physicians’ diagnostic workup of and ED collaboration in general are widely unclear. Furthermore, previous research has indicated that CDDS usage results in increased diagnostic investigations and higher costs.^20–22^

Furthermore, considering rare DDx suggested by CDDS for patients presenting with common symptoms may trigger extensive (and expensive) additional testing that would not have been conducted otherwise. Whether possible benefits justify the potentially increasing costs in our healthcare system remains to be determined. Currently, evidence to inform such a debate is lacking.

### Aims

In this study, we aim (1) to assess the effect of the DDx generator usage on diagnostic quality and patient outcome in patients admitted to EDs, (2) to understand the influence that DDx generators have on the physicians’ diagnostic workflow and the workup in the EDs and (3) to investigate the effect of the DDx generator on resource utilisation and costs.

## Methods

We will conduct a multicentre, outcome assessor and patient-blinded, cluster-randomised, multiperiod crossover superiority trial in four Swiss EDs.

**Figure 1:**
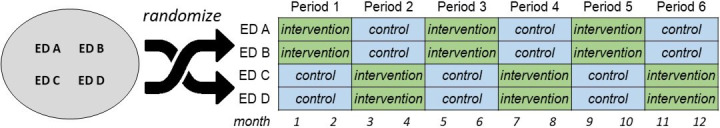
Study design. ED, emergency department.

The participating EDs will be randomly allocated to two different sequences of alternating intervention and control periods of 2 months each (figure 1). During the intervention periods, physicians of the respective EDs will have access to the DDx generator under investigation (Isabel Pro by Isabel Healthcare) and will be asked to consult the CDDS at least once during diagnostic workup. During the control periods, the DDx generator will not be accessible to the physicians and the diagnostic process will follow usual care. No wash-out periods will be applied as no substantial cross-over effects are expected given the nature of the intervention.

The interventional trial will be accompanied by a qualitative substudy. First, observations (step 1) will be performed. They will also be used to guide questions for interviews. If needed, a focus group (step 2) will support this process. Subsequently, semistructured interviews (step 3) will be performed with physicians during an intervention phase. Observation will be repeated in a later period to assess changes in workflow, satisfaction change, etc, and if necessary, interviews will be performed again (step 4). The qualitative part observes the physicians diagnostic process, not the patient and the focus of the present manuscript is on the interventional trial.

### Study sample

Patient subjects fulfilling all of the following inclusion criteria are eligible for the investigation:

- Informed consent signed by the subject (see online supplemental file 1).
- Presentation to the ED with either fever, abdominal pain, syncope or a non-specific complaint (NSC) as chief complaint. All these complaints occur frequently, can result from a large number of underlying diseases, and thus provide room for diagnostic error. Furthermore, there are no universally agreed algorithms for the diagnostic workup of any of these symptoms (as there is for chest pain, for example). NSC is defined in this study as all chief complaints not included in the checklist of specific complaints according to Nemec *et al*.^23^
- Triaged as ‘no acute life-threatening condition’ because study inclusion would otherwise not be feasible in many cases.
- The study subject is 18 years old or older.

10.1136/bmjopen-2023-072649.supp1 Supplementary data

The presence of any one of the following exclusion criteria, patients will not be eligible for study inclusion:

- Trauma as chief complaint, because there are standardised diagnostic workups, most trauma patients receive radiographic imaging and the potential benefit of a DDx generator is questionable.
- Pregnancy (self-reported), because options for diagnostic workup are severely constrained in these patients, and presentation is mostly related to pregnancy and its complications, reducing room for error to occur and be remediated.
- Worsening of a known pre-existing condition or medical referral with a definite diagnosis, because the diagnosis is clear in this case.
- Inability to follow the informed consent and investigation procedures, for example, due to language barriers, psychological disorder, admittance via police, detainee status.
- Previous enrolment into the current investigation.

Patients presenting to any of the participating EDs during the study period between June 2022 and June 2023 will be registered in the EHR system and triaged by hospital staff according to clinical routine. A dedicated and trained study nurse will consecutively screen the EHR for eligible study patients. Eligible patients will be informed about the nature of the study and written informed consent will be obtained. All consenting and included study patients will be allocated automatically to the investigation group of the respective site during the respective period.

### Outcomes

An advisory board composed of subject-matter experts and three patient representatives have advised on outcome selection, measurement and prioritisation.

The primary outcome is a binary score indicating the presence or absence of ‘risk to diagnostic quality’, defined as one or more of the following:

- Death within 14 days after ED discharge (yes/no).
- Unscheduled medical care (ED revisits, general practicioner (GP) visits or hospitalisation) within 14 days after ED discharge (yes/no).
- Unexpected intensive care unit admission from ward within 24 hours of hospitalisation (yes/no).
- Diagnostic discrepancy between the ED discharge diagnosis and the latest diagnosis 14 days after ED admission (yes/no).

The primary endpoint is positive, if one or multiple of the criteria above are true, and false if none of the criteria above occur.

Secondary outcomes are:

- All variables that compose the primary endpoint separately.
- Unscheduled ED/GP revisits after 72 hours and 7 days.
- Length of stay in the ED in hours.
- Length of hospital stay if hospitalised.
- Diagnostic tests conducted in the ED.
- Diagnostic tests after ED discharge.
- Resource consumption in the ED (costs).
- Care consumption after ED discharge.
- Discharge destination.
- Number and disease groups of DDx provided by physicians.
- Number of cases where the computer-generated DDx list entails the diagnosis on day 14.
- Diagnostic error based on full chart review for a random subset of patients.
- CDDS usage (timing and number of queries).

Additional outcomes are physician confidence calibration, advice-seeking behaviour and collaboration assessed by physician observations, interviews and focus groups to understand how DDx generator affects diagnostic workflow in the ED and physicians’ advice seeking, collaboration and confidence calibration. Table 1 provides an overview of methods used for outcome assessment. The full-visit structure of the study can be found in the online supplemental file 2.

10.1136/bmjopen-2023-072649.supp2 Supplementary data

**Table 1:** Primary and secondary outcomes and their method of measurement

Primary outcome	
Parameter	Data collection
Death within 14±4 days after ED discharge	Study nurses will review the EHR and contact the patient and GP by phone for vital status information after 14 days.
Unscheduled medical care for the same complaints within 14±4 days after ED discharge	Study nurses will contact patients by phone 14 days after discharge to collect information about follow-up medical care and diagnosis. GP or other medical care provider will be contacted when patients indicate a visit there.
IMC unit admission within 24 hours after ED discharge if hospitalised	Retrieved from the EHR system.
Discrepancy in ED discharge diagnosis and diagnosis 14±4 days after ED discharge	ED discharge differential diagnoses will be looked up in the ED discharge letter and registered in the study database by a study nurse. Study nurses will contact patients by phone 14 days after discharge to collect information about follow-up medical care and diagnosis. Additionally, medical records from the local hospital and the GP are reviewed. Information about the current differential diagnoses from the EHR or the GP (if available) is preferred over patient’s self-reported diagnosis. Two blinded physicians will independently review the ED differential diagnoses from ED discharge and at day 14 and define whether there is a discrepancy based on clinical judgement.
Secondary outcomes	
Number and type of diagnostic tests performed in the ED (and hospital if hospitalised)	Study nurses will retrieve the type and number of diagnostic tests performed during ED (and hospital) stay from the EHR. The following diagnostic tests (yes/no) will be recorded: Lab (blood), Lab (urine), Lab (sputum), MRI, CT, sonography, X-ray, other. Additionally, the number and type of blood samples taken and consultation of specialists will be recorded.
Resource consumption during ED (and hospital if hospitalised) stay.	Resource consumption in Swiss Francs for each patient (costs of diagnostic tests, consultations, personal and material caused) will be obtained from the hospital’s administrative database to assess costs caused during the ED stay and costs caused during hospital stay if hospitalised. Costs will additional be itemised according to the hospital’s cost centres.
Sick leave days	Sick leave days will be converted to Swiss francs through the average labour productivity database of OECD.
Physician confidence in ED diagnosis	Study nurses hand out an online questionnaire to the diagnosing resident physicians after patient discharge.
Discharge destination	Retrieved from EHR.
ED LOS (hours)	Retrieved from EHR.
Hospital LOS if hospitalised (days)	Retrieved from EHR.
CDDS usage (number of queries)	Monitored by the CDDS and stored automatically in the eCRF via API.
CDDS input and output data	Monitored by the CDDS and stored automatically in the eCRF via API.
Patient-reported outcomes	Study nurses will conduct patient interviews by phone after 14 days.
Diagnostic error	Chart review will be performed for a random sample of 50 patients with a positive primary endpoint and 50 patients with a negative primary endpoint to seek for diagnostic errors using the Revised Safer Dx Instrument according to Singh *et al*.^26 27^
Number and type of diagnostic tests performed in the ED (and hospital if hospitalised)	Study nurses will retrieve the type and number of diagnostic tests performed during ED (and hospital) stay from the EHR. The following diagnostic tests (yes/no) will be recorded: Lab (blood), Lab (urine), Lab (sputum), MRI, CT, sonography, X-ray, other. Additionally, the number of blood samples and the number of specialist consultations will be recorded.

API, Application Programming Interface; CDDS, computerised diagnostic decision support systems; eCRF, electronic case report form; ED, emergency department; EHR, electronic health record; GP, General practitioner; LOS, Length of stay; OECD, Organisation for Economic Co-operation and Development.

### Follow-up

Patients discharged home will be contacted and interviewed via phone by a designated and trained study nurse 14 days after the first ED visit. If patients report any medical care consumption after discharge, the corresponding medical institution (GP, hospital, etc) will be contacted to obtain the medical record. For patients who are hospitalised at follow-up, the relevant data at the time of follow-up will be obtained from the EHR system.

### Blinding

Treating physicians cannot be blinded towards the patients’ study allocation for obvious reasons. Patients will be blinded, that is, they will not be informed about the current condition (intervention or control period) of the ED they present to. Also, study nurses conducting follow-up interviews with patients and their general practitioners will be blinded and all raters involved in the study will be blinded (when determining whether a diagnostic discrepancy occurred and when conducting chart review to validate the measure of our primary outcome).

### Randomisation

Participating EDs were randomly assigned to one of the two sequences by an independent, blinded person before the start of the recruitment phase using concealed envelopes. Patients enrolled during intervention periods of the respective site will be allocated to the intervention group or to the control group if the site is in a control phase at time of enrolment.

### Study intervention

The medical device software under investigation is the CDDS ‘Isabel Pro—the DDx Generator’ from ISABEL Healthcare. The German language version will be used in the present study. A systematic review and meta-analysis found that Isabel Pro was associated with the highest accuracy of the suggested diagnoses among all investigated CDDS (pooled rate=0.89, 95% CI=0.83 to 0.94; I^2^=82%, p<0.001).^17^ Isabel Pro is simple to use and time to enter data and obtain diagnostic suggestions takes less than a minute.^24^ Additional factors supporting the choice of Isabel Pro as DDx generator for our study is the facilitated integration in the workflow management software and the available German interface.

Isabel Pro has been developed for health professionals, that is, the software is intended to support clinicians in broadening their differentials in the diagnostic workup. Namely, users are provided with a list of potential DDx based on patient characteristics and key symptoms entered as free text. Usage of the software itself does not require extensive training. Isabel healthcare provides training videos of 3–5 min length and 2–3 slides with tips for usage. We additionally provide a short training to the German interface. The most important usage instructions are also provided on the web interface of the software itself. All residents will additionally be briefed by a designated study nurse before first usage. Study nurses are monitoring adherence to the protocol and remind physicians to use the CDDS if necessary.

### Sample size

The sample size calculation has been performed for a multiperiod cross-over cluster randomised controlled trial according to Hemming *et al* using the Shiny CRT Calculator.^25^ The trial is designed to have a power of 80% to detect a clinically significant between-condition difference in the primary outcome of 5% points on an alpha level of 0.05.

For the primary outcome, we assumed a positive composite score in 12% of the cases in the control condition.^4^ Further assumptions were a cross-sectional sampling and exchangeable correlation structure, an intracluster correlation between 0.01 and 0.05, a coefficient of variation of cluster size of 0.5 and a 10% lost to follow-up patients. Minimal sample size under the conditions above is 1184 patients in total. The sample size calculation was initially performed for four periods; however, the trial has been extended to six periods during study conduct due to slow recruitment.

### Statistical analysis

Statistical analysis will be based on generalised linear mixed models (GLMM) using appropriate post hoc techniques (eg, for subgroup analyses).

Standard descriptive statistics and illustrative graphing will be used throughout, along with normality testing (eg, Shapiro-Wilk) in order to check assumptions for the appropriate use of parametric testing approaches. Transformations to normality for variables not fulfilling normality assumptions will be considered (eg, log, Box-Cox, etc), while non-parametric testing using counterparts of ad-hoc parametric procedures will also be an option as needed (eg, Kruskal-Wallis instead of one-way analysis of variance, the latter being part of the generalised linear model family). The R Language for Statistical Programming (R Foundation for Statistical Computing, Vienna, Austria) will be used for data analysis. A test-wise two-sided p value of less than 0.05 (after post-hoc and/or false discovery rate adjustment if deemed appropriate) will be considered statistically significant.

Data will be analysed according to the intention-to-treat principle. Data from all participants with or without protocol violation including dropouts and withdrawals will be included in the main analysis. A per-protocol (PP) analysis will be performed as sensitivity analysis. Patients from the intervention group will be removed from the PP analysis if no CDDS query has been documented and vice versa, patients from the control group will be removed from the analysis if physicians self-report the query of any DDx generator outside the study protocol.

For the primary outcome (presence or no presence of a positive diagnostic quality risk score), a GLMM with a binomial distribution family and exchangeable correlation structure will be performed. The GLMM takes into account a random intercept for each site, resident and attending physician. Diagnosing resident and attending physicians are nested within sites. The condition (intervention and control) and the period (periods 1–6) will be included as fixed factors under the assumption of equality of carryover effects. Additionally, presenting chief complaint, patient’s age, sex and comorbidity index will be added as covariates.

For all secondary endpoints, summary statistics appropriate to the distribution will be tabulated by treatment group. Secondary efficacy analyses will parallel the primary analysis.

More details on statistical analyses are described in the statistical analysis plan, which will be published together with the full clinical investigational protocol and other study relevant documentation on Zenodo.org and referred to on clinicaltrials.gov and digitaldiagnosis.ch.

### Monitoring

An experienced study nurse from another clinic not involved in the study will be assigned for monitoring to check for trial documentation and source data verification. No specific audits or inspections are planned. An interim analysis for safety outcomes is planned after the end of the second period. Based on their evaluations, it will be decided by the sponsor-investigator and the local principal investigators if premature stopping of the clinical investigation is required.

### Patient and public involvement

To ensure practical relevance of this project, applicability of its findings and dissemination of its results to foster implementation, we have established two study advisory boards. An expert board assembles specialists of the various areas affected by CDDS, while on the patient board, the perspective of patients and the public is represented. Both boards have separately met to advise us on the outcome measures for this project, the criteria for CDDS selection and potential strategies for dissemination. The advisory board will meet again after results of the study are available to foster dissemination and implementation. The expert board and network of the coinvestigators further ensures a continuous flow of information to relevant stakeholders.

## Ethics and dissemination

ClinicalTrials.gov Identifier: NCT05346523. The study was reviewed and approved by the cantonal ethics committee of canton Berne under Project-ID 2022-D0002 and Swissmedic, the Swiss national regulatory authority on medical devices. Any protocol modification will be communicated with the local PIs and study teams and approved by the ethics and Swissmedic.

Patient risk due to study participation is minimal. Informed patient consent will be obtained before patient inclusion. A patient advisory board was consulted during study design and substantially contributed to the definition of primary and secondary study outcomes.

Insights provided by this study will be disseminated to scientists, healthcare professionals, study participants, patient societies, industry and policy-makers. Data will be submitted for publication in internationally peer-reviewed scientific journals. The privacy of each subject and confidentiality of their information shall be preserved in reports and publication of data. Minimal coded subject-level datasets and statistical codes will be published in an online repository together with the corresponding publications.

## Supplementary Material

Reviewer comments

Author's manuscript
