# Supplementary material for: Effects of a computerised diagnostic decision support tool on diagnostic quality in emergency departments: study protocol of the DDx-BRO multicentre cluster randomised cross-over trial
Source: BMJ Open. 2023 Mar 28;13(3):e072649. doi: 10.1136/bmjopen-2023-072649 (PMC10069571; doi:10.1136/bmjopen-2023-072649)
Supplement: Supplementary data [file bmjopen-2023-072649supp001.pdf]

INSELSPITAL
UNIVERSITÄTSSPITAL BERN
HÔPITAL UNIVERSITAIRE DE BERNE

Universitäres NOTFALL ZENTRUM Erwachsene

Direktor und Chefarzt
Prof. Dr. med. Aristomenis Exadaktylos

Leitender Arzt Forschung
Prof. Dr. med. Wolf Hautz, MME

Anfrage zur Teilnahme an medizinischer Forschung:

Auswirkungen einer Diagnose-Support-Software auf die Diagnosequalität bei Notfall-Patienten

Sehr geehrte Dame, sehr geehrter Herr

Wir fragen Sie hier an, ob Sie bereit wären, an unserem Forschungsvorhaben mitzuwirken.

Ihre Teilnahme ist freiwillig. Alle Daten, die in diesem Projekt erhoben werden, unterliegen strengen Datenschutzvorschriften. Das Forschungsvorhaben wird durchgeführt von der Insel Gruppe. Bei Interesse informieren wir Sie gerne über die Ergebnisse aus dem Forschungsvorhaben.

In einem Gespräch erklären wir Ihnen die wichtigsten Punkte und beantworten Ihre Fragen. Damit Sie sich bereits jetzt ein Bild machen können, hier das Wichtigste vorweg. Im Anschluss folgen dann weitere, detaillierte Informationen.

Warum führen wir dieses Forschungsvorhaben durch?

- Kommen Patienten mit unspezifischen Symptomen (Beschwerden) in den Notfall, kommen oftmals eine Vielzahl von Diagnosen in Frage. Für die Diagnosestellung ist es wichtig, neben der hauptsächlich vermuteten Erkrankungen auch an alternative Diagnosen, sogenannte Differentialdiagnosen, zu denken.
  Dabei kann es vorkommen, dass wichtige Differentialdiagnosen vergessen gehen oder nicht in Betracht gezogen werden.

- Heutzutage gibt es Computerprogramme die Ärzte und Ärztinnen bei der Diagnosestellung unterstützen sollen. Die Fachpersonen geben relevante Daten wie Symptome in das Computerprogramm ein, welches diese dann mit einer grossen Datenbank abgleicht und passende Diagnosen vorschlägt.

- In unserem Forschungsvorhaben wollen wir herausfinden, ob sich die Diagnosequalität verbessert, wenn die behandelnden Ärzte und Ärztinnen im Notfallzentrum eine solches Computerprogramm verwenden.

Was muss ich bei einer Teilnahme tun? – Was geschieht mit mir bei einer Teilnahme?

- Ablauf: Wenn Sie sich entscheiden mitzumachen, wird der behandelnde Assistenzarzt oder die behandelnde Assistenzärztin ihre Symptome in das Computerprogramm eingeben, sofern zu dieser Zeit das Computerprogramm zur

Marcin T, *et al. BMJ Open* 2023; 13:e072649. doi: 10.1136/bmjopen-2023-072649

Verfügung steht. Das Ärzteteam entscheidet dann, ob und welche der vorgeschlagenen Differentialdiagnosen in Betracht gezogen und unter Umständen weiter abgeklärt werden sollten. Steht das Programm zu dieser Zeit nicht zur Verfügung, erfolgt die Diagnose wie üblich ohne Hilfe durch ein Computerprogramm.

- Aufwand: 14 Tage nach ihrem Notfallaufenthalt werden wir Sie telefonisch kontaktieren um mit Ihnen ein kurzes Gespräch über ihren gesundheitlichen Verlauf zu führen. Dieses Gespräch dauert ungefähr 10-20 Minuten. Ebenfalls kann ihr Hausarzt kontaktiert werden um Informationen zum gesundheitlichen Verlauf zu erhalten.

Welcher Nutzen und welches Risiko sind damit verbunden?

Nutzen

- Sie helfen mit Ihrer Teilnahme künftigen Patientinnen und Patienten.

Risiko und Belastung

- Ein direktes Risiko oder Belastung durch die Verwendung des Computerprogrammes besteht nicht. Allerdings könnte es vorkommen, dass die Ärzte und Ärztinnen zusätzliche Abklärungen vornehmen möchten um vorgeschlagene Diagnosen ausschliessen zu können. Die Verantwortung, dass keine unnötigen Abklärungen vorgenommen werden liegt nach wie vor bei den Fachpersonen und ihrer medizinischen Einschätzung.

Mit Ihrer Unterschrift am Ende des Dokuments bezeugen Sie, dass Sie freiwillig teilnehmen und dass Sie die Inhalte des gesamten Dokuments verstanden haben.

Marcin T, et al. BMJ Open 2023; 13:e072649. doi: 10.1136/bmjopen-2023-072649

Detaillierte Information

1. Ziel und Auswahl

Unser Forschungsvorhaben bezeichnen wir in dieser Informationsschrift als Studie. Wenn Sie an dieser Studie teilnehmen, sind Sie eine *Studienteilnehmerin* bzw. ein *Studienteilnehmer*.

Bevor ein Medizinprodukt angewendet wird, muss es bei Studienteilnehmenden wissenschaftlich untersucht werden. Wir wollen daher untersuchen, welche Auswirkungen es auf die Diagnosequalität im Notfall hat, wenn Ärzte und Ärztinnen die Medizinprodukte-Software (*Isabel – Der DDx Generator von Isabel Healthcare Ltd*.) als Unterstützung bei der Diagnosestellung verwenden.

Die Software (Computerprogramm) hat zum Ziel, Symptome und andere Patientendaten mit einer großen Datenbank an Diagnosen abzugleichen um den Fachpersonen passende Diagnosen vorzuschlagen. Die Software schlägt keine definitiven Diagnosen vor, sondern soll die Fachpersonen anregen Diagnosen in Betracht zu ziehen, welche Ihnen ansonsten entgangen wären.

Die Software schlägt gemäß durchgeführten Studien sehr oft die richtige Diagnose vor und wird bereits seit einigen Jahren in diversen Spitälern im englischsprachigem Raum verwendet. Welche Auswirkungen die Anwendung der Software auf die Diagnosestellung in Notfall-Kliniken hat wurde aber bislang noch nicht systematisch mit einer Studie untersucht.

Wir fragen Sie an, da alle Personen teilnehmen können, die mit Fieber, Bauchschmerzen, kurzzeitiger Bewusstlosigkeit oder unspezifischen Symptomen auf den Notfall kommen und nicht akut lebensbedrohlich gefährdet sind.

2. Allgemeine Informationen

- Wir wissen noch wenig über die tatsächlichen Auswirkungen, wenn Ärzte und Ärztinnen auf dem Notfall ein Computerprogramm verwenden um nach möglichen Diagnosen zu suchen.
- Wir möchten daher herausfinden, ob die Nutzung einer solchen Software die Diagnosequalität und Patientensicherheit verbessert, ob diagnostische Abklärungen zu- oder abnehmen und ob sich die Zusammenarbeit zwischen den Fachpersonen verändert.
- Die Software wird den teilnehmenden Spitälern über mehrere Zeiträume abwechslungsweise zur Verfügung gestellt (Interventionsperiode) respektive enthalten (Kontrollperiode). Wenn Sie während einem Zeitraum auf den Notfall kommen, wo die Software zur Verfügung steht, gehören Sie zur Interventionsgruppe, ansonsten zur Kontrollgruppe.
  Gehören Sie zur Interventionsgruppe werden die behandelnden Assistenzärztinnen und Assistenzärzte aufgefordert, Ihre Symptome in das Computerprogramm einzugeben um mögliche Diagnosen nachzuschlagen.
  Gehören Sie zur Kontrollgruppe haben die Assistenzärzte keinen Zugriff auf das Programm und die Diagnosestellung folgt der üblichen Behandlung. Sie erhalten keine Informationen in welche Gruppe Sie zugeteilt werden.
- Der Hersteller und die zuständige Behörde haben die Software bis zur letzten Änderung der Medizinprodukte-Regulierung nicht als Medizinprodukt klassifiziert. Aus diesem Grund hat die Software keine Medizinprodukte-Zertifizierung, wurde aber bereits auf den Markt gebracht und in Gesundheitseinrichtungen an Patienten angewendet.
- Insgesamt nehmen vier Notfallzentren aus der Region Bern und Solothurn teil. Die Dauer der Studie beträgt 8 bis 12 Monate, je nachdem wie schnell wir eine ausreichende Anzahl an Patientinnen und Patienten für diese Studie gewinnen können. Während dieser Zeit sollen insgesamt ungefähr 1‘200 Patienten und Patientinnen eingeschlossen werden.
- Wir machen diese Studie so, wie es die Gesetze in der Schweiz vorschreiben. Außerdem beachten wir alle international anerkannten Richtlinien. Die zuständige Ethikkommission und Swissmedic haben die Studie geprüft und bewilligt.

- Eine Beschreibung dieser Studie finden Sie auch auf der Internetseite des Bundesamtes für Gesundheit unter www.kofam.ch

3. Ablauf

- Die studienbedingte Intervention (Abfrage der Diagnose-Software) erfolgt durch die behandelnden Ärzte. Sie werden davon nichts bemerken.
- Für Sie entsteht aufgrund der Studienteilnahme kein zusätzlicher Aufwand während Ihrem Notfallaufenthalt. Untersuchungen und Behandlungen erfolgen nur aufgrund medizinischer Notwendigkeit, basierend auf der Einschätzung und Verordnung ihres behandelnden Ärzteteams, im Notfallzentrum.
- Außerhalb der klinischen Routine möchten wir gerne wissen, wie es Ihnen in den 14 Tagen seit der Behandlung auf dem Notfall ergangen ist. Dazu würde Sie ein Mitarbeiter oder eine Mitarbeiterin des Studienteams der Insel Gruppe nach 14 Tagen anrufen um ein telefonisches Gespräch von rund 10-20 Minuten mit Ihnen zu führen.
- Ihre Hausärztin/Ihr Hausarzt wird über Ihre Studienteilnahme informiert. Es kann sein, dass wir Ihre Hausärztin/Ihr Hausarzt nach 14 Tagen kontaktieren um im Rahmen der Studie zusätzliche Angaben zu ihrem gesundheitlichen Verlauf zu erhalten.
- Sie werden vorzeitig aus der Studie ausgeschlossen, wenn die sichere Durchführung der Studie nicht gewährleistet werden kann und gemäß Studienteam oder Prüfperson in ihrem medizinischem Interesse liegt oder Sie selbst den Abbruch der Studie wünschen.

4. Nutzen

Es kann sein, dass die Teilnahme keinen direkten Nutzen für Sie mit sich bringt. Durch die Teilnahme an dieser Studie helfen Sie uns aber zu verstehen, ob generell die Diagnosequalität im klinischen Alltag auf dem Notfallzentrum mit einer Diagnosesoftware verbessert werden kann.

5. Freiwilligkeit und Pflichten

Sie nehmen freiwillig teil. Wenn Sie nicht an dieser Studie teilnehmen oder später Ihre Teilnahme zurückziehen wollen, müssen Sie dies nicht begründen. Ihre medizinische Behandlung/Betreuung ist unabhängig von Ihrem Entscheid gewährleistet.

Wenn Sie an dieser Studie teilnehmen, werden Sie gebeten:

- Das Studienteam beim Telefoninterview nach 14 Tagen über den Verlauf der Erkrankung zu informieren und neue Diagnosen, Symptome und Beschwerden sowie Änderungen im Befinden zu melden.

6. Risiken und Belastungen

Es kann sein, dass Ärzte und Ärztinnen durch das Computerprogramm auf mögliche Diagnosen hingewiesen werden, welche sie ansonsten nicht berücksichtigt hätten. Dies kann dazu führen, dass weiterführende Abklärungen veranlasst werden um diese Diagnosen auszuschließen und Ihr Aufenthalt auf dem Notfall verlängert wird. Diagnostische Abklärungen werden aber nur aus medizinischen Gründen durchgeführt, das heißt, wenn das Ärzteteam die weiteren Abklärungen auch als medizinisch relevant erachtet. Das Risiko für unbegründete Abklärungen ist somit gering.

7. Alternativen

Die Teilnahme an der Studie ist mit Chancen und Risiken verbunden. Wenn Sie der Studie nicht einwilligen, erhalten Sie die normale Behandlung im Notfall. Es kann aber nicht ausgeschlossen werden, dass ihre behandelnden Ärzte trotzdem ein alternatives, öffentlich zugängliches Computerprogramm innerhalb der Routinebehandlung verwenden um nach möglichen Diagnosen zu suchen.

8. Ergebnisse

Es gibt

1. individuelle Ergebnisse der Studie, die Sie direkt betreffen,
2. objektive End-Ergebnisse der gesamten Studie.

Marcin T, et al. BMJ Open 2023; 13:e072649. doi: 10.1136/bmjopen-2023-072649

Zu 1: Die Prüfärztin/der Prüfarzt wird Sie im Verlauf der Studie über alle für Sie persönlich wichtigen, neuen Ergebnisse und Erkenntnisse informieren. Sie werden mündlich und schriftlich informiert und können dann erneut entscheiden, ob Sie an der Studie weiter teilnehmen möchten.
Zu 2: Die objektiven End-Ergebnisse der gesamten Studie werden der fachwissenschaftlichen Öffentlichkeit zugängig gemacht und der Schweizer Aufsichtsbehörde SwissMedic und den zuständigen Ethikkomissionen zugestellt. Die Veröffentlichung, respektive Weitergabe der Ergebnisse erfolgt in verschlüsselter und/oder zusammengefasster Form, in der kein Rückschluss auf Sie als einzelnen Patienten möglich ist. Ihr Prüfärztin/Ihr Prüfarzt kann Ihnen am Ende der Studie eine Zusammenfassung der Gesamtergebnisse zukommen lassen, wenn Sie dies wünschen.

9. Vertraulichkeit von Daten

Für diese Studie werden Daten zu Ihrer Person und Gesundheit erfasst und bearbeitet, teilweise in automatisierter Form. Bei der Datenerhebung werden Ihre Daten verschlüsselt. Verschlüsselung bedeutet, dass alle Bezugsdaten, die Sie identifizieren könnten (Name, Geburtsdatum etc.), gelöscht und durch einen Code ersetzt. Personen, die keinen Zugang zu dieser Schlüssel-Liste haben, können keine Rückschlüsse auf Ihre Person ziehen. Die Schlüssel-Liste wird nach Abschluss der Studie von einer Person ausserhalb des Studienteam (Universitätsklinik für Intensivmedizin, Insel Gruppe) aufbewahrt.
Nur sehr wenige Fachpersonen werden Ihre unverschlüsselten Daten sehen und zwar nur, um Aufgaben im Rahmen der Studie zu erfüllen. Diese Personen unterliegen der Schweigepflicht. Sie als teilnehmende Person haben das Recht auf Einsicht in Ihre Daten.

Alle Vorgaben des Datenschutzes werden streng eingehalten. Es ist möglich, dass Ihre Daten in verschlüsselter Form, zum Beispiel für eine Publikation, übermittelt werden müssen und anderen Forschern zur Verfügung gestellt werden können (auch ins Ausland).
Ärztinnen und Ärzte, die für die Nachbehandlung verantwortlich sind, können kontaktiert werden, um Auskunft über Ihren Gesundheitszustand zu geben.

Diese Studie kann durch die zuständige Ethikkommission, die Arzneimittelbehörde Swissmedic oder durch den Sponsor-Investigator, der die Studie veranlasst hat, überprüft werden. Die Prüfarztin/der Prüfarzt muss dann Ihre Daten für solche Kontrollen offenlegen. Alle müssen absolute Vertraulichkeit wahren.

10. Rücktritt
Sie können jederzeit von der Studie zurücktreten. Die bis dahin erhobenen Datenwerden in diesem Fall allerdings noch verschlüsselt ausgewertet.
Im Falle eines Rücktritts bleiben Ihre Daten weiterhin verschlüsselt in den Studiendokumenten. Dies dient vorrangig Ihrer medizinischen Sicherheit. Prüfen Sie bitte, ob Sie damit einverstanden sind, bevor Sie bei der Studie mitmachen.

11. Entschädigung
Wenn Sie an dieser Studie teilnehmen, bekommen Sie dafür keine finanzielle Entschädigung. Es entstehen Ihnen oder Ihrer Krankenkasse keine Kosten durch die Teilnahme.
Die Ergebnisse dieser Studie können unter Umständen dazu beitragen, kommerzielle Produkte (z.B. Medizinprodukte-Software) zu entwickeln. Durch Ihre Studienteilnahme haben Sie kein Anrecht auf Anspruch an kommerziellen Entwicklungen (z.B. Patente).

12. Haftung
Die Insel Gruppe die die Studie veranlasst hat und für die Durchführung verantwortlich ist, haftet für Schäden, die Ihnen im Zusammenhang mit dem Prüfprodukt entstehen könnten. Die Voraussetzungen und das Vorgehen dazu sind gesetzlich geregelt.

Marcin T, *et al. BMJ Open* 2023; 13:e072649. doi: 10.1136/bmjopen-2023-072649

Die Insel Gruppe hat daher eine Versicherung bei der *Zürich Versicherungs-Gesellschaft AG, Thurgauerstrasse 80, 8050 Zürich* abgeschlossen, um in einem möglichen Schadenfall für die Haftung aufkommen zu können.
Sollten Sie durch die Teilnahme an dieser Studie einen Schaden erleiden, so wenden Sie sich bitte an die Prüfärztin/den Prüfarzt oder an das oben erwähnte Versicherungsunternehmen.

13. Finanzierung
Die Studie wird mehrheitlich durch den Schweizerischen Nationalfonds 407740_187284 / 1 bezahlt.

14. Kontaktperson(en)
Sie dürfen jederzeit Fragen zur Studienteilnahme stellen. Auch bei Unsicherheiten oder Notfällen, die während der Studie oder danach auftreten, wenden Sie sich bitte an:

Prof. Dr. med. Wolf Hautz
Leitender Arzt Forschung
Universitäres Notfallzentrum
Inselspital Bern
Freiburgstrasse 16C, 3010 Bern
Tel.: +41 31 632 57 01
E-Mail: wolf.hautz@insel.ch

Oder an den Dienstoberarzt des Notfallzentrums
Tel.-Nr.: +41 31 632 24 02 (365 Tage, 24h besetzt)

Marcin T, *et al. BMJ Open* 2023; 13:e072649. doi: 10.1136/bmjopen-2023-072649

INSELSPITAL
UNIVERSITÄTSSPITAL BERN
HÔPITAL UNIVERSITAIRE DE BERNE

Universitäres NOTFALL ZENTRUM Erwachsene

Einwilligungserklärung

Schriftliche Einwilligungserklärung zur Teilnahme an einer klinischen Studie
Bitte lesen Sie dieses Formular sorgfältig durch. Bitte fragen Sie, wenn Sie etwas nicht verstehen oder wissen möchten. Für die Teilnahme ist Ihre schriftliche Einwilligung notwendig.

**BASEC-Nummer (nach Einreichung):**	2022-D0002
**Titel der Studie (wissenschaftlich und Laiensprache)**:	Auswirkungen einer Diagnose-Support-Software auf die Diagnosequalität bei Notfall-Patienten
**Verantwortliche Institution (Sponsor-Investigator mit Adresse)**:	Prof. Dr. med. Wolf Hautz<br>Leitender Arzt Forschung<br>Universitäres Notfallzentrum<br>Inselspital Bern<br>Freiburgstrasse 16C, 3010 Bern<br>Tel.: +41 31 632 57 01
**Ort der Durchführung**:	Universitäres Notfallzentrum<br>Inselspital Bern<br>Telefon: +41 31 632 24 02
**Prüfärztin/Prüfarzt am Studienort:**<br>Name und Vorname in Druckbuchstaben:	Prof. Dr. Wolf Hautz<br>Leitender Arzt Forschung
**Teilnehmerin/Teilnehmer:**<br>Name und Vorname in Druckbuchstaben:<br>Geburtsdatum:	☐ weiblich ☐ männlich

- Ich wurde von dem oder der unterzeichnenden Prüfarzt/Prüfärztin oder der delegierten Person mündlich und schriftlich über den Zweck, den Ablauf der Studie mit der Prüfsoftware über mögliche Vor- und Nachteile sowie über eventuelle Risiken informiert.
- Ich nehme an dieser Studie freiwillig teil und akzeptiere den Inhalt der mir ausgehändigten schriftlichen Information. Ich hatte genügend Zeit, meine Entscheidung zu treffen.
- Meine Fragen im Zusammenhang mit der Teilnahme an dieser Studie sind mir beantwortet worden. Ich behalte die schriftliche Information und erhalte eine Kopie meiner schriftlichen Einwilligungserklärung.
- Ich wurde über mögliche Alternativen zur Studie, dem normalen Standardverfahren auf dem Notfall aufgeklärt.
- Ich bin einverstanden, dass meine Hausärztin/mein Hausarzt über meine Teilnahme an der Studie informiert wird.
- Im Fall einer Weiterbehandlung ausserhalb des Prüfzentrums ermächtige ich meine nachbehandelnden Ärztinnen und Ärzte, meine für die Studie relevanten Daten der Prüfärztin/dem Prüfarzt zu übermitteln.
- Ich bin einverstanden, dass die zuständigen Fachleute des Sponsor-Investigators, der zuständigen Ethikkommission und der Arzneimittelbehörde Swissmedic zu Prüf- und Kontrollzwecken in meine unverschlüsselten Daten Einsicht nehmen dürfen, jedoch unter strikter Einhaltung der Vertraulichkeit.
- Bei Ergebnissen, die direkt meine Gesundheit betreffen, werde ich informiert. Wenn ich das nicht wünsche, informiere ich meine Prüfärztin/ meinen Prüfarzt.

- Ich weiss, dass meine gesundheitsbezogenen und persönlichen Daten nur in verschlüsselter Form zu Forschungszwecken für diese Studie weitergegeben werden können (auch ins Ausland). Der Sponsor gewährleistet, dass der Datenschutz nach Schweizer Standard eingehalten wird.
- Ich kann jederzeit und ohne Angabe von Gründen von der Studienteilnahme zu rücktreten. Meine weitere medizinische Behandlung ist unabhängig von der Studienteilnahme gewährleistet. Die bis zum Rücktritt erhobenen Daten werden noch im Rahmen der Studie ausgewertet.
- Ich bin darüber informiert, dass die Insel Gruppe eine Versicherung abgeschlossen hat, welche Schäden, die auf das Forschungsprojekt zurückzuführen sind, deckt.
- Ich bin mir bewusst, dass die in der Informationsschrift genannten Pflichten einzuhalten sind. Im Interesse meiner Gesundheit kann mich die Prüfärztin/der Prüfarzt jederzeit von der Studie ausschliessen.

Ort, Datum	Unterschrift Teilnehmerin/Teilnehmer

Bestätigung der Prüfärztin/des Prüfarztes bzw. der Prüfperson: Hiermit bestätige ich, dass ich dieser Teilnehmerin/diesem Teilnehmer Wesen, Bedeutung und Tragweite der Studie erläutert habe. Ich versichere, alle im Zusammenhang mit dieser Studie stehenden Verpflichtungen gemäss in der Schweiz geltenden Rechts zu erfüllen. Sollte ich im Verlauf der Studie von Aspekten erfahren, welche die Bereitschaft der Teilnehmerin/des Teilnehmers zur Studienteilnahme beeinflussen könnten, werde ich sie/ihn umgehend darüber informieren.

Ort, Datum	Name und Vorname der Prüfärztin/des Prüfarztes bzw. der Prüfperson in Druckbuchstaben<br><br>Unterschrift der Prüfärztin/des Prüfarztes

Marcin T, *et al. BMJ Open* 2023; 13:e072649. doi: 10.1136/bmjopen-2023-072649
