# Supplementary material for: Effects of a computerised diagnostic decision support tool on diagnostic quality in emergency departments: study protocol of the DDx-BRO multicentre cluster randomised cross-over trial
Source: BMJ Open. 2023 Mar 28;13(3):e072649. doi: 10.1136/bmjopen-2023-072649 (PMC10069571; doi:10.1136/bmjopen-2023-072649)
Supplement: Supplementary data [file bmjopen-2023-072649supp002.pdf]

INVESTIGATION SCHEDULE

Investigation Periods	Screening	Consent (ICF)	Treatment, Intervention Period		Follow-up
Emergency Care in the emergency room	Admittance and Triage	Waiting Time	Medical examination and treatment		discharged or hospitalized
Time	-1 to -5h	-1 to -5h	0 to 1h	1h + LOS	14d±4d
**A)** Enrolment					
In- /Exclusion Criteria	x				
Patient information		x			
Patient consent (ICF)		x			
**B)** Intervention					
CDDS application during intervention period			x	(x)	
CDDS usage monitoring by study nurses			x		
**C) Assessments** (CR: performed within clinical routine as appropriate)					
Demographics	x				
Chief complaint	CR				
Triage assessment	CR				
Medical history			CR		
Physical examination			CR	CR	
Vital signs			CR	CR	
Laboratory tests			CR	CR	
Other diagnostic tests			CR	CR	
CDDS input / output data collection			x	(x)	
Physician questionnaire			x	(x)	
Patient telephone interviews					x
Medical record review from ER, hospital and/or GP			x	x	x
Serious Adverse Events, Adverse device effects			x	x	x
Device Deficiencies			x	x	
**D) Primary Outcome Score**					
All-cause mortality					x
Unscheduled medical care if discharged (GP, ER revisit, hospitalization)					x
Unexpected ICU admission within 24h if hospitalized					x
Current diagnosis for presenting complaint				x	x

**E) Secondary Outcomes**					
Number and cost of ER diagnostic tests				x	x
Time to ER diagnosis				x	
ER differential diagnoses				x	
Physician confidence in ER diagnosis				x	
Discharge destination				CR	
ER LOS				CR	
Hospital LOS if hospitalized					CR
CDDS usage (number of queries)			x	(x)	
Patient reported outcomes					x
ER, Emergency room; ICU, intensive care unit; CDDS, computerized diagnostic decision support system; GP, general practitioner; LOS, length of stay; CR, clinical routine					
