# Supplementary material for: Effects of a computerised diagnostic decision support tool on diagnostic quality in emergency departments: study protocol of the DDx-BRO multicentre cluster randomised cross-over trial
Source: BMJ Open. 2023 Mar 28;13(3):e072649. doi: 10.1136/bmjopen-2023-072649 (PMC10069571; doi:10.1136/bmjopen-2023-072649)
Supplement: Author's manuscript [file bmjopen-2023-072649.draft_revisions.pdf]

BMJ Open

BMJ Open is committed to open peer review. As part of this commitment we make the peer review history of every article we publish publicly available.

When an article is published we post the peer reviewers' comments and the authors' responses online. We also post the versions of the paper that were used during peer review. These are the versions that the peer review comments apply to.

The versions of the paper that follow are the versions that were submitted during the peer review process. They are not the versions of record or the final published versions. They should not be cited or distributed as the published version of this manuscript.

BMJ Open is an open access journal and the full, final, typeset and author-corrected version of record of the manuscript is available on our site with no access controls, subscription charges or pay-per-view fees (http://bmjopen.bmj.com).

If you have any questions on BMJ Open's open peer review process please email info.bmjopen@bmj.com

BMJ Open

Effects of a computerized diagnostic decision support tool on diagnostic quality in emergency departments – study protocol of the DDX-BRO multi-centre cluster randomized cross-over trial

Journal:	*BMJ Open*
Manuscript ID	bmjopen-2023-072649
Article Type:	Protocol
Date Submitted by the Author:	10-Feb-2023

Complete List of Authors:	Marcin, Thimo; Inselspital University Hospital Bern, Department of Emergency Medicine<br>Hautz, Stefanie C.; Inselspital University Hospital Bern, Emergency medicine<br>Singh, Hardeep; Michael E DeBakey VA Medical Center, Center for Innovations in Quality, Effectiveness and Safety (IQuESt), ; Baylor College of Medicine, Department of Medicine<br>Zwaan, Laura; Erasmus Medical Center, Institute of Medical Education Research Rotterdam (iMERR)<br>Schwappach, David; University of Bern, Institute of Social and Preventive Medicine (ISPM)<br>Krummrey, Gert; Inselspital University Hospital Bern, Department of Emergency Medicine; Bern University of Applied Sciences<br>Schauber, Stefan; University of Oslo, CEMO & HUS<br>Nendaz , Mathieu; University of Geneva, Department of medicine<br>Exadaktylos, Aristomenis; Inselspital University Hospital Bern, Department of Emergency Medicine<br>Müller, Martin; Inselspital University Hospital Bern, Department of Emergency Medicine<br>Lambrigger, Cornelia; Inselspital University Hospital Bern, Department of Emergency Medicine<br>Sauter, Thomas; Inselspital University Hospital Bern, Department of Emergency Medicine<br>Lindner, Gregor; Burgerspital Solothurn, Department of Internal and Emergency Medicine<br>Bosbach, Simon; Insel Gruppe AG, Spital Tiefenau<br>Griesshammer, Ines; Insel Gruppe AG, Spital Muensingen<br>Hautz, Wolf; Inselspital University Hospital Bern, Department of Emergency Medicine

Keywords:	ACCIDENT & EMERGENCY MEDICINE, Health informatics < BIOTECHNOLOGY & BIOINFORMATICS, Information management < BIOTECHNOLOGY & BIOINFORMATICS, Decision Making, Quality in health care < HEALTH SERVICES ADMINISTRATION & MANAGEMENT, INTERNAL MEDICINE

Title:

Effects of a computerized diagnostic decision support tool on diagnostic quality in emergency departments – study protocol of the DDX-BRO multi-centre cluster randomized cross-over trial.

Authors:

Thimo Marcin[1], Stefanie C Hautz[1], Hardeep Singh[2], Laura Zwaan[3], David Schwappach[4], Gert Krummrey[1,5], Stefan K Schauber[6],Mathieu Nendaz[7], Aristomenis Exadaktylos[1], Martin Müller[1], Cornelia Lambrigger[1], Thomas C Sauter[1], Gregor Lindner[1,8], Simon Bosbach[9], Ines Griesshammer[8,10], Wolf E Hautz[1]

[1] Department of Emergency Medicine, Inselspital University Hospital Bern, University of Bern, Bern, Switzerland
[2] Baylor college of Medicine, Housten, TX, USA
[3] iMED, EDASMUS, The Netherlands
[4] Institute of Social and Preventive Medicine (ISPM), University of Bern, Bern, Switzerland
[5] Bern University of Applied Sciences, Biel, Switzerland
[6] CEMO & HUS, University of Oslo, Norway
[7] Department of medicine, University of Geneva, Switzerland
[8] Bürgerspital Solothurn, Switzerland
[9] Spital Tiefenau, Inselgruppe AG, Bern, Switzerland
[10] Spital Münsingen, Inselgruppe AG, Switzerland

Correspondence: Wolf Hautz, M.D., M.M.E., Department of Emergency Medicine, Inselspital University Hospital Bern, Freiburgstrasse, 3010 Bern, Switzerland. wolf.hautz@insel.ch

Based on clinical investigation protocol, v.1.5, 10/10/2022

For peer review only - http://bmjopen.bmj.com/site/about/guidelines.xhtml

Abstract:

Introduction: Misdiagnosis occurs in about 5% of emergency department (ED) patients. Computerized diagnostic decision support systems (CDDS) suggesting differential diagnoses (DDx) to physicians aim to improve clinical reasoning and diagnostic quality. However, controlled clinical trials investigating their effectiveness and safety are absent and consequences of its use in clinical practice unknown. We aim to investigate the effect of CDDS use by ED physicians on diagnostic quality, workflow, resource consumption and patient outcomes.

Methods and analysis: This is a multicentre, outcome assessor and patient blinded, cluster-randomised, multi-period crossover superiority trial. A validated differential diagnosis generator will be implemented in four EDs and randomly allocated to a sequence of six alternating intervention and control periods. During intervention periods, the treating ED physician will be asked to consult the CDDS at least once during diagnostic workup. During control periods, physicians will not have access to the CDDS and diagnostic workup will follow usual clinical care. Key inclusion criteria will be patients' presentation to the ED with either fever, abdominal pain, syncope or a non-specific complaint as chief complaint. Primary outcome is a binary diagnostic quality risk score composed of presence of an unscheduled medical care after discharge, change in diagnosis or death during time of follow-up or an unexpected upscale in care within 24h after hospital admission. Time of follow-up is 14 days. At least 1184 patients will be included.

Secondary outcomes will include additional patient related outcomes such as length of hospital stay, diagnostics and costs as well as data regarding CDDS usage. A qualitative sub-study will provide additionally insights to physicians' confidence calibration and diagnostic workflow.

Statistical analysis will use general linear mixed modelling methods.

Ethics and dissemination: ClinicalTrials.gov Identifier: NCT05346523. Approved by the cantonal ethics committee of canton Berne (2022-D0002) and Swissmedic, the Swiss national regulatory authority on medical devices.

For peer review only

Strength and limitations:

- Study addresses diagnostic error in emergency medicine, a significant patient safety topic which is under-researched
- First prospective randomized clinical trial on the effect of computerized diagnostic decision support programs in a real-world setting
- Patient relevant outcomes, including mortality and unscheduled revisits
- Multi-centre cluster randomized crossover trial with blinded outcome assessment
- Non-random selection of participating emergency rooms

Introduction

Diagnostic error in emergency medicine

Getting the right diagnosis in health care is key to provide an explanation of a patient's health problem and informs subsequent health care and treatment.[1] Diagnostic errors have been estimated to occur in about 5% of outpatient and emergency department (ED) patients, sometimes with devastating medical and economic consequences.[2,3] We have previously found clinically significant diagnostic discrepancies between ED and hospital discharge diagnosis in 12.3% of the patients, which were associated with longer hospital stay and increased mortality.[4] The causes for diagnostic error can be diverse, but one major cause is human error.[1,3,5] This includes the consideration of incomplete patient histories, failure to consider alternative diagnoses, lack of knowledge and lack of recognition of clinical findings by physicians.[5–9] An accurate diagnosis is the basis for all treatment and care and hence, improving the diagnostic process and accuracy is key to improving patient safety and outcome.

Clinical decision support systems

Within the last decades, digitalization in health care has led to a rapid evolution of various clinical decision support systems (CDS) supposed to augment clinicians in their complex decision-making processes.[10] A recent systematic literature review on electronic health record (EHR) integrated generic CDS in the ED found positive effects on various outcomes in 83% of the included articles.[11] However, they included various CDS types with heterogeneous targets and measured outcomes and studies of mixed quality. CDS that were built to support clinicians specifically in the process of diagnosis (computerized diagnostic decision support system; CDDS) have not shown such promising results as other types of CDS so far, mostly due to negative physician perception and biases, poor accuracy or poor system integration.[10]

Differential diagnosis

A key role in the diagnostic process has been attributed to differential diagnoses (DDx) as they guide physicians in considering or excluding possible diagnoses in the ongoing diagnostic process.[12] Research on diagnostic decision making found that in absence of a correct diagnostic hypothesis, physicians tried to explain evidence away that did not fit their diagnosis and that misdiagnosis occurred most when the correct diagnosis was not even considered.[13,14] Broadening the differentials has been consistently recognized as an important measure to avoid diagnostic errors.[15,16] But given the vast amount of diseases and clinical manifestations that ED physicians are confronted with, they cannot be expected to think of all possible DDx. It is likely that they either do not recall or know all of the potential DDx fitting the clinical presentation and symptoms of any given patient.

For peer review only - http://bmjopen.bmj.com/site/about/guidelines.xhtml

DDx Generators

Reminding physicians of potential DDx is the aim of DDx generators. DDx generators are built to provide physicians with a list of DDx based on clinical data input, such as patient characteristics, symptoms, findings and other factors, and were first developed in the 1970s. Since then, DDx generators have evolved in the last decades as computational methods have advanced. Nowadays, DDx generators are capable of matching input data to large electronic databases of diagnoses using varying computational methods such as Bayesian probabilities or text mining techniques,[17] and to subsequently retrieve the correct diagnosis with a acceptable accuracy. Likewise, integration in EHR systems has improved continuously and enhanced usability in clinical practice.[10]

Accuracy of DDx generators

A systematic review and meta-analysis from 2016 investigating the efficacy and utility of DDx generators found a pooled accuracy of computer suggested diagnoses of 0.7, meaning that the correct diagnosis was among the suggested DDx in 70% of the cases. However, only small improvements were seen in before and after studies, where clinicians had the opportunity to revisit their diagnoses following a DDx generator consultation.[17] However, most of the included studies were considered to be at high risk for selection, funding and publication bias and the findings of some older included studies may not be applicable to the new generation of DDx generators, given their improved accuracy and usability. Most importantly, DDx generators were applied retrospectively across the majority of the studies, reducing the external validity of the results.

Effectiveness of DDx generators and knowledge gaps

To the best of our knowledge, studies investigating the effectiveness and safety of DDx generators in ‘real world’ clinical setting are absent. Therefore, it is unknown to what extent the use of these CDDS actually improves the quality of medical diagnoses and consecutive health outcomes of the individual patient. In addition, research indicates that collaboration among clinicians is frequent and substantially improves diagnostic accuracy.[1,18,19] CDDS may lower ED physician’s perception whether they need advice from other team members and thus reduce the likelihood of collaboration. The effect of a DDx generator on physicians’ diagnostic workup of and ED collaboration in general are widely unclear. Furthermore, previous research has indicated that CDDS usage results in increased diagnostic investigations and higher costs.[20–22]

Furthermore, considering rare differential diagnoses suggested by CDDS for patients presenting with common symptoms may trigger extensive (and expensive) additional testing that would not have been conducted otherwise. Whether possible benefits justify the potentially increasing costs in our health care system remains to be determined. Currently, evidence to inform such a debate is lacking.

Aims

In this study, we aim 1) to assess the effect of the DDx generator usage on diagnostic quality and patient outcome in patients admitted to EDs, 2) to understand the influence that DDx generators have on the physicians’ diagnostic workflow and the workup in the emergency departments and 3) to investigate the effect of the DDx generator on resource utilization and costs.

For peer review only - http://bmjopen.bmj.com/site/about/guidelines.xhtml

Methods

We will conduct a multi-centre, outcome assessor and patient blinded, cluster-randomized, multi-period crossover superiority trial in 4 Swiss EDs.

The participating EDs will be randomly allocated to two different sequences of alternating intervention and control periods of two months each (figure 1). During the intervention periods, physicians of the respective EDs will have access to the DDx generator under investigation (Isabel Pro by Isabel Healthcare Ltd) and will be asked to consult the CDDS at least once during diagnostic workup. During the control periods, the DDx generator will not be accessible to the physicians and the diagnostic process will follow usual care. No wash-out periods will be applied as no substantial cross-over effects are expected given the nature of the intervention.

Study sample

Patient subjects fulfilling all of the following inclusion criteria are eligible for the investigation:

- Informed Consent signed by the subject
- Presentation to the ED with either fever, abdominal pain, syncope or a non-specific complaint (NSC) as chief complaint. All these complaints occur frequently, can result from a large number of underlying diseases and thus provide room for diagnostic error. Furthermore, there are no universally agreed algorithms for the diagnostic workup of any of these symptoms (as there is for chest pain, for example). NSC are defined in this study as all chief complaints not included in the check list of specific complaints according to Nemec et al..[23]
- Triaged as “no acute life-threatening condition” because study inclusion would otherwise not be feasible in many cases.
- The study subject is 18 years old or older.

The presence of any one of the following exclusion criteria, patients will not be eligible for study inclusion:

- Trauma as chief complaint, because there are standardized diagnostic workups, most trauma patients receive radiographic imaging and the potential benefit of a DDx generator is questionable.
- Pregnancy (self-reported), because options for diagnostic workup are severely constrained in these patients, and presentation is mostly related to pregnancy and its complications, reducing room for error to occur and be remediated.
- Worsening of a known pre-existing condition or medical referral with a definite diagnosis, because the diagnosis is clear in this case.
- Inability to follow the informed consent and investigation procedures, e.g., due to language barriers, psychological disorder, admittance via police, detainee status.
- Previous enrolment into the current investigation.

Patients presenting to any of the participating EDs during the study period between June 2022 and June 2023 will be registered in the electronic health record system (EHR) and triaged by hospital staff according to clinical routine. A dedicated and trained study nurse will consecutively screen the EHR for eligible study patients. Eligible patients will be informed about the nature of the study and written informed consent will be obtained. All consenting and included study patients will be allocated automatically to the investigation group of the respective site during the respective period.

For peer review only - http://bmjopen.bmj.com/site/about/guidelines.xhtml

Outcomes

An advisory board composed of subject matter experts and three patient representatives will advise on outcome selection, measurement and prioritisation.

Primary outcome is a binary score indicating the presence or absence of "risk to diagnostic quality", defined asone or more of the following:

- death within 14 days after ED discharge (yes/no)
- unscheduled medical care (ED revisits, GP visits or hospitalization) within 14 days after ED discharge (yes/no)
- unexpected intensive care unit admission from ward within 24h of hospitalization (yes/no)
- diagnostic discrepancy between the ED discharge diagnosis and the latest diagnosis 14 days after ED admission (yes/no)

The primary endpoint is positive, if one or multiple of the criteria above are true, and negative if none of the criteria above occur.

Secondary outcomes are

- all variables that compose the primary endpoint separately
- unscheduled ED/GP revisits after 72h and 7 days
- length of stay in the ED in hours
- length of hospital stay if hospitalized
- diagnostic tests conducted in the ED
- diagnostic tests after ED discharge
- resource consumption in the ED (costs)
- Care consumption after ED discharge
- discharge destination
- patient reported outcomes
- number and disease groups of DDx provided by physicians
- number of cases where the computer generated DDx list entails the diagnosis on day 14
- diagnostic error based on full chart review for a random subset of patients
- CDDS usage (timing and number of queries)

Additional outcomes are physician confidence calibration, advice seeking behaviour and collaboration assessed by physician observations, interviews and focus groups to understand how DDx generator affect diagnostic workflow in the ED and physicians' advice seeking, collaboration and confidence calibration. Table 1 provides an overview of methods used for outcome assessment. The full visit structure of the study can be found in a supplemental information.

For peer review only - http://bmjopen.bmj.com/site/about/guidelines.xhtml

Table 1: Primary and secondary outcomes and their method of measurement

**Primary outcome**	
**Parameter**	**Data collection**
Death within 14±4 days after ED discharge	Study nurses will review the EHR and contact the patient and GP by phone for vital status information after 14 days.
Unscheduled medical care for the same complaints within 14±4 days after ED discharge	Study nurses will contact patients by phone 14 days after discharge to collect information about follow-up medical care and diagnosis. GP or other medical care provider will be contacted when patients indicate a visit there.
IMC unit admission within 24h after ED discharge if hospitalized	Retrieved form the EHR system.
Discrepancy in ED discharge diagnosis and diagnosis 14±4 days after ED discharge	ED discharge differential diagnoses will be looked up in the ED discharge letter and registered in the study database by a study nurse. Study nurses will contact patients by phone 14 days after discharge to collect information about follow-up medical care and diagnosis. Additionally, medical records from the local hospital and the GP are reviewed. Information about the current differential diagnoses from the EHR or the GP (if available) are preferred over patient's self-reported diagnosis.<br>Two blinded physicians will independently review the ED differential diagnoses from ED discharge and at day 14 and define whether there is a discrepancy based on clinical judgement.
**Secondary outcomes**	
Number and type of diagnostic tests performed in the ED (and hospital if hospitalized)	Study nurses will retrieve the type and number of diagnostic tests performed during ED (and hospital) stay from the EHR. The following diagnostic tests (yes/no) will be recorded: Lab (Blood), Lab (Urine), Lab (Sputum), MRI, CT, Sonography, X-ray, other.<br>Additionally, the number and type of blood samples taken and consultation of specialists will be recorded.
Resource consumption during ED (and hospital if hospitalized) stay.	Resource consumption in Swiss Francs (CHF) for each patient (costs of diagnostic tests, consultations, personal and material caused) will be obtained from the hospitals administrative database to assess costs caused during the ED stay and costs caused during hospital stay if hospitalized. Costs will additional be itemized according to the hospital's cost centres.
Sick leave days	Sick leave days will be converted to Swiss francs (CHF) through the average labour productivity database of OECD
Physician confidence in ED diagnosis	Study nurses hand out an online questionnaire to the diagnosing resident physicians after patient discharge.
Discharge destination	Retrieved from EHR
ED LOS [hours]	Retrieved from EHR
Hospital LOS if hospitalized [days]	Retrieved from EHR
CDDS usage (number of queries)	Monitored by the CDDS and stored automatically in the electronic case report form (eCRF) via API.
CDDS in- and output data	Monitored by the CDDS and stored automatically in the eCRF via API.
Patient reported outcomes	Study nurses will conduct patient interviews by phone after 14 days
Diagnostic Error	Chart review will be performed for a random sample of 50 patients with a positive primary endpoint and 50 patients with a negative primary endpoint to seek for diagnostic errors using the Revised Safer Dx Instrument according to Singh et al..[24,25]
Number and type of diagnostic tests performed in the ED (and hospital if hospitalized)	Study nurses will retrieve the type and number of diagnostic tests performed during ED (and hospital) stay from the EHR. The following diagnostic tests (yes/no) will be recorded:<br>Lab (Blood), Lab (Urine), Lab (Sputum), MRI, CT, Sonography, X-ray, other.

For peer review only - http://bmjopen.bmj.com/site/about/guidelines.xhtml

	Additionally, the number of blood samples and the number of specialist consultations will be recorded.

Follow-up

Patients discharged home will be contacted and interviewed via phone by a designated and trained study nurse 14 days after the first ED visit. If patients report any medical care consumption after discharge, the corresponding medical institution (GP, hospital etc.) will be contacted to obtain the medical record. For patients who are hospitalized at follow-up, the relevant data at the time of follow-up will be obtained from the EHR system.

Blinding

Treating physicians cannot be blinded towards the patients' study allocation for obvious reasons. Patients will be blinded, i.e., they will not be informed about the current condition (intervention or control period) of the ED they present to. Also, study nurses conducting follow up interviews with patients and their general practitioners will be blinded and all raters involved in the study will be blinded (when determining whether a diagnostic discrepancy occurred and when conducting chart review to validate the measure of our primary outcome).

Randomization

Participating EDs were be randomly assigned to one of the two sequences by an independent, blinded person before start of the recruitment phase using concealed envelopes. Patients enrolled during intervention periods of the respective site will be allocated to the intervention group or to the control group if the site is in a control phase at time of enrollment.

Study intervention

The medical device software under investigation is the CDDS "Isabel Pro - the DDx Generator" from ISABEL Healthcare Ltd. The German language version will be used in the present study. A systematic review and meta-analysis found that Isabel Pro was associated with the highest accuracy of the suggested diagnoses among all investigated CDDS (pooled rate = 0.89, 95% CI = 0.83 to 0.94; I2 = 82%, p<0.001).[17] Isabel Pro is simple to use and time to enter data and obtain diagnostic suggestions takes less than a minute.[26] Additional factors supporting the choice of Isabel Pro as DDx Generator for our study is the facilitated integration in the workflow management software and the available German interface.

Isabel Pro is developed for health professionals, i.e., the software is intended to support clinicians in broadening their differentials in the diagnostic workup. Namely, users are provided with a list of potential DDx based on patient characteristics and key symptoms entered as free text. Usage of the software itself does not require extensive training. Isabel healthcare provides training videos of 3-5 minutes length and 2-3 slides with tips for usage. We additionally provide a short training to the German interface. The most important usage instructions are also provided on the web interface of the software itself. All residents will additionally be briefed by a designated study nurse before first usage. Study nurses are monitoring adherence to the protocol and remind physicians to use the CDDS if necessary.

For peer review only - http://bmjopen.bmj.com/site/about/guidelines.xhtml

Sample Size

The sample size calculation has been performed for a multi-period cross-over cluster randomized controlled trial according to Hemming at al. (2020) using The Shiny CRT Calculator.[27] The trial is designed to have a power of 80% to detect a clinically significant between-condition-difference in the primary outcome of 5 percent points on an alpha level of 0.05.

For the primary outcome, we assumed a positive composite score in 12% of the cases in the control condition.[4] Further assumptions were a cross-sectional sampling and exchangeable correlation structure, an intra cluster correlation between 0.01 and 0.05, a coefficient of variation of cluster size of 0.5 and a 10% lost-to-follow up patients. Minimal sample size under the conditions above is 1'184 patients in total. The sample size calculation was initially performed for 4 periods, however, the trial has been extended to 6 periods during study conduct due to slow recruitment.

Statistical Analysis

Statistical analysis will be based on general linear mixed modelling (GLMM) methods using appropriate post hoc techniques (e.g for subgroup analyses).

Standard descriptive statistics, and illustrative graphing will be used throughout, along with normality testing (e.g. Shapiro-Wilk) in order to check assumptions for the appropriate use of parametric testing approaches. Transformations to normality for variables not fulfilling normality assumptions will be considered (e.g. log, Box-Cox etc.), while nonparametric testing using counterparts of ad-hoc parametric procedures will also be an option as needed (e.g. Kruskal-Wallis instead of one-way ANOVA, the latter being part of the GLM family). The R Language for Statistical Programming (R Foundation for Statistical Computing, Vienna, Austria) will be used for data analysis. A test-wise 2-sided p-value of less than 0.05 (after post-hoc and/or FDR adjustment if deemed appropriate) will be considered statistically significant.

Data will be analysed according to the intention-to-treat principle. Data from all participants with or without protocol violation including dropouts and withdrawals will be included in the main analysis. A Per-Protocol (PP) analysis will be performed as sensitivity analysis. Patients from the intervention group will be removed from the PP-analysis if no CDDS query has been documented and vice versa, patients from the control group will be removed from the analysis if physicians self-report the query of any DDx generator outside the study protocol.
For the primary outcome (presence or no presence of a positive diagnostic quality risk score), a generalized linear mixed model (GLMM) with a binomial distribution family and exchangeable correlation structure will be performed. The GLMM takes into account a random intercept for each site, resident and attending physician. Diagnosing resident and attending physicians are nested within sites. The condition (intervention and control) and the period (period 1 to 6) will be included as fixed factors under the assumption of equality of carry-over effects. Additionally, presenting chief complaint, patient's age, sex and comorbidity index will be added as covariates.

For all secondary endpoints, summary statistics appropriate to the distribution will be tabulated by treatment group. Secondary efficacy analyses will parallel the primary analysis. More details on statistical analyses are described in the statistical analysis plan published on clinicaltrials.gov and digitaldiagnosis.ch

For peer review only - http://bmjopen.bmj.com/site/about/guidelines.xhtml

For peer review only

Patient and Public Involvement

To ensure practical relevance of this project, applicability of its findings and dissemination of its results to foster implementation, we have established two study advisory boards. An expert board assembles specialists of the various areas affected by CDDS, while on the patient board, the perspective of patients and the public is represented. Both boards have separately met to advise us on the outcome measures for this project, the criteria for CDDS selection and potential strategies for dissemination. The advisory board will be meet again after results of the study are available to foster dissemination and implementation. The expert board and network of the co-investigators further ensures a continuous flow of information to relevant stakeholders.

Ethics and dissemination

ClinicalTrials.gov Identifier: NCT05346523. The study was reviewed and approved by the cantonal ethics committee of canton Berne under Project-ID 2022-D0002 and Swissmedic, the Swiss national regulatory authority on medical devices.

Patient risk due to study participation is minimal. Informed patient consent will be obtained before patient inclusion. A patient advisory board was consulted during study design and substantially contributed to the definition of primary and secondary study outcomes.

Insights provided by this study will be disseminated to scientists, health care professionals, study participants, patient societies, industry and policymakers. Data will be submitted for publication in internationally peer-reviewed scientific journals. The privacy of each subject and confidentiality of their information shall be preserved in reports and publication of data. Minimal coded subject-level datasets and statistical codes will be published in an online repository together with the corresponding publications.

Authors contributions

WEH and TM wrote the first draft of the study protocol and of this manuscript. All authors provided critical revisions and important comments on the initial draft. All authors approved of the final version of the protocol and of this manuscript. WEH obtained funding for the study. AE, GL, SB and IG conduct the study at the emergency departments they chair.

Funding

This work is supported by the Swiss National Science Foundation under its National Research Program NRP77, grant number 187284.

Competing interests

WEH has received research funding from the European Union, the Swiss National Science Foundation, Zoll foundation, Dräger Medical Germany, Mundipharma Research UK, MDI International Australia, Roche Diagnostics Germany, all outside the submitted work. WEH has provided paid consultancies to AO foundation Switzerland and MDI International Australia, all outside the submitted work. WEH has received financial support for a congress he chaired from EBSCO Germany, Isabel Healthcare UK, Mundipharma Medical Switzerland, VisualDx USA, all outside the submitted work.

For peer review only

For peer review only - http://bmjopen.bmj.com/site/about/guidelines.xhtml

References

1 Committee on Diagnostic Error in Health Care, Board on Health Care Services, Institute of Medicine, *et al. Improving Diagnosis in Health Care*. Washington, D.C.: : National Academies Press 2015. doi:10.17226/21794

2 Singh H, Meyer AND, Thomas EJ. The frequency of diagnostic errors in outpatient care: estimations from three large observational studies involving US adult populations. *BMJ Qual Saf* 2014;**23**:727–31. doi:10.1136/bmjqs-2013-002627

3 Newman-Toker DE, Peterson SM, Badihian S, *et al. Diagnostic Errors in the Emergency Department: A Systematic Review*. Rockville (MD): : Agency for Healthcare Research and Quality (US) 2022. http://www.ncbi.nlm.nih.gov/books/NBK588118/ (accessed 24 Jan 2023).

4 Hautz WE, Kämmer JE, Hautz SC, *et al.* Diagnostic error increases mortality and length of hospital stay in patients presenting through the emergency room. *Scand J Trauma Resusc Emerg Med* 2019;**27**:54. doi:10.1186/s13049-019-0629-z

5 Zwaan L, de Bruijne M, Wagner C, *et al.* Patient Record Review of the Incidence, Consequences, and Causes of Diagnostic Adverse Events. *ARCH INTERN MED* 2010;**170**:7.

6 Graber ML, Franklin N, Gordon R. Diagnostic Error in Internal Medicine. *Arch Intern Med* 2005;**165**:1493. doi:10.1001/archinte.165.13.1493

7 Croskerry P. The importance of cognitive errors in diagnosis and strategies to minimize them. *Acad Med* 2003;**78**:775–80.

8 Norman GR, Monteiro SD, Sherbino J, *et al.* The Causes of Errors in Clinical Reasoning: Cognitive Biases, Knowledge Deficits, and Dual Process Thinking. *Acad Med* 2017;**92**:23–30. doi:10.1097/ACM.0000000000001421

9 Zwaan L, Thijs A, Wagner C, *et al.* Relating Faults in Diagnostic Reasoning With Diagnostic Errors and Patient Harm: *Acad Med* 2012;**87**:149–56. doi:10.1097/ACM.0b013e31823f71e6

10 Sutton RT, Pincock D, Baumgart DC, *et al.* An overview of clinical decision support systems: benefits, risks, and strategies for success. *NPJ Digit Med* 2020;**3**:17. doi:10.1038/s41746-020-0221-y

11 Patterson BW, Pulia MS, Ravi S, *et al.* Scope and Influence of Electronic Health Record–Integrated Clinical Decision Support in the Emergency Department: A Systematic Review. *Ann Emerg Med* Published Online First: January 2019. doi:10.1016/j.annemergmed.2018.10.034

12 Jain B. The key role of differential diagnosis in diagnosis. *Diagnosis* 2017;**4**:239–40. doi:10.1515/dx-2017-0005

13 Kostopoulou O. Diagnosis of Difficult Cases in Primary Care. *J Health Serv Res Policy* 2010;**15**:71–4. doi:10.1258/jhsrp.2009.09s112

14 Kostopoulou O, Devereaux-Walsh C, Delaney BC. Missing Celiac Disease in Family Medicine: The Importance of Hypothesis Generation. *Med Decis Making* 2009;**29**:282–90. doi:10.1177/0272989X08327493

15 Singh H, Giardina TD, Meyer AND, *et al.* Types and Origins of Diagnostic Errors in Primary Care Settings. *JAMA Intern Med* 2013;**173**:418. doi:10.1001/jamainternmed.2013.2777

For peer review only - http://bmjopen.bmj.com/site/about/guidelines.xhtml

16 Ely JW, Kaldjian LC, D'Alessandro DM. Diagnostic Errors in Primary Care: Lessons Learned. *J Am Board Fam Med* 2012;**25**:87–97. doi:10.3122/jabfm.2012.01.110174

17 Riches N, Panagioti M, Alam R, *et al.* The Effectiveness of Electronic Differential Diagnoses (DDX) Generators: A Systematic Review and Meta-Analysis. *PLOS ONE* 2016;**11**:e0148991. doi:10.1371/journal.pone.0148991

18 Hautz WE, Kammer JE, Schauber SK, *et al.* Diagnostic performance by medical students working individually or in teams. *JAMA* 2015;**313**:303–4. doi:10.1001/jama.2014.15770

19 Kämmer JE, Hautz WE, Herzog SM, *et al.* The Potential of Collective Intelligence in Emergency Medicine: Pooling Medical Students' Independent Decisions Improves Diagnostic Performance. *Med Decis Making* 2017;**37**:715–24. doi:10.1177/0272989X17696998

20 Semigran HL, Linder JA, Gidengil C, *et al.* Evaluation of symptom checkers for self diagnosis and triage: audit study. *BMJ* 2015;:h3480. doi:10.1136/bmj.h3480

21 Kanagasingam Y, Xiao D, Vignarajan J, *et al.* Evaluation of Artificial Intelligence–Based Grading of Diabetic Retinopathy in Primary Care. *JAMA Netw Open* 2018;**1**:e182665. doi:10.1001/jamanetworkopen.2018.2665

22 Apkon M. A Randomized Outpatient Trial of a Decision-Support Information Technology Tool. *Arch Intern Med* 2005;**165**:2388. doi:10.1001/archinte.165.20.2388

23 Nemec M, Koller MT, Nickel CH, *et al.* Patients presenting to the emergency department with non-specific complaints: the Basel Non-specific Complaints (BANC) study. *Acad Emerg Med Off J Soc Acad Emerg Med* 2010;**17**:284–92. doi:10.1111/j.1553-2712.2009.00658.x

24 Singh H, Bradford A, Goeschel C. Operational measurement of diagnostic safety: state of the science. *Diagnosis* 2021;**8**:51–65. doi:10.1515/dx-2020-0045

25 Singh H, Khanna A, Spitzmueller C, *et al.* Recommendations for using the Revised Safer Dx Instrument to help measure and improve diagnostic safety. *Diagnosis* 2019;**6**:315–23. doi:10.1515/dx-2019-0012

26 Graber ML, Mathew A. Performance of a web-based clinical diagnosis support system for internists. *J Gen Intern Med* 2008;**23 Suppl 1**:37–40. doi:10.1007/s11606-007-0271-8

27 Hemming K, Kasza J, Hooper R, *et al.* A tutorial on sample size calculation for multiple-period cluster randomized parallel, cross-over and stepped-wedge trials using the Shiny CRT Calculator. *Int J Epidemiol* 2020;**49**:979–95. doi:10.1093/ije/dyz237

Figure Legends

Figure 1: Study Design

Word count

3286

Acknowledgements

Isabel Healthcare Ltd provided technical support to the study team in the usage of the Isabel Pro API. They further provided free exploratory access to their English as well as German API during trial setup and supported study implementation with the provision of educational material. Lastly, Isabel

For peer review only - http://bmjopen.bmj.com/site/about/guidelines.xhtml

provided technical documentation to facilitate the regulatory evaluation of the study. Dr. Andreas Limacher of CTU Bern provided advice on sample size calculation. We are grateful to the subject matter experts and patient representatives who participated in the advisory board of the study.

For peer review only

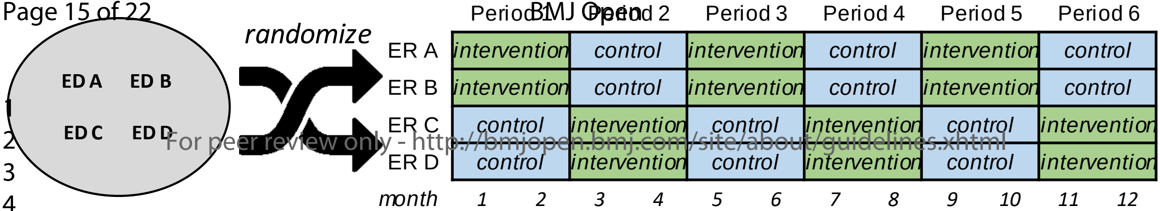

For peer review only - http://bmjopen.bmj.com/site/about/guidelines.xhtml

INVESTIGATION SCHEDULE

Investigation Periods	Screening	Consent (ICF)	Treatment, Intervention Period		Follow-up
Emergency Care in the emergency room	Admittance and Triage	Waiting Time	Medical examination and treatment		discharged or hospitalized
Time	-1 to -5h	-1 to -5h	0 to 1h	1h + LOS	14d±4d
**A)** Enrolment					
In- /Exclusion Criteria	x				
Patient information		x			
Patient consent (ICF)		x			
**B)** Intervention					
CDDS application during intervention period			x	(x)	
CDDS usage monitoring by study nurses			x		
C) **Assessments** (CR: performed within clinical routine as appropriate)					
Demographics	x				
Chief complaint	CR				
Triage assessment	CR				
Medical history			CR		
Physical examination			CR	CR	
Vital signs			CR	CR	
Laboratory tests			CR	CR	
Other diagnostic tests			CR	CR	
CDDS input / output data collection			x	(x)	
Physician questionnaire			x	(x)	
Patient telephone interviews					x
Medical record review from ER, hospital and/or GP			x	x	x
Serious Adverse Events, Adverse device effects			x	x	x
Device Deficiencies			x	x	
**D) Primary Outcome Score**					
All-cause mortality					x
Unscheduled medical care if discharged (GP, ER revisit, hospitalization)					x
Unexpected ICU admission within 24h if hospitalized					x
Current diagnosis for presenting complaint				x	x

For peer review only

**E) Secondary Outcomes**					
Number and cost of ER diagnostic tests				x	x
Time to ER diagnosis				x	
ER differential diagnoses				x	
Physician confidence in ER diagnosis				x	
Discharge destination				CR	
ER LOS				CR	
Hospital LOS if hospitalized					CR
CDDS usage (number of queries)			x	(x)	
Patient reported outcomes					x

ER, Emergency room; ICU, intensive care unit; CDDS, computerized diagnostic decision support system; GP, general practitioner; LOS, length of stay; CR, clinical routine

For peer review only

For peer review only

SPIRIT
STANDARD PROTOCOL ITEMS: RECOMMENDATIONS FOR INTERVENTIONAL TRIALS

SPIRIT 2013 Checklist: Recommended items to address in a clinical trial protocol and related documents*

Section/item	Item No	Description	Addressed on page number
**Administrative information**			
Title	1	Descriptive title identifying the study design, population, interventions, and, if applicable, trial acronym	1
Trial registration	2a	Trial identifier and registry name. If not yet registered, name of intended registry	2
	2b	All items from the World Health Organization Trial Registration Data Set	-
Protocol version	3	Date and version identifier	1
Funding	4	Sources and types of financial, material, and other support	12
Roles and responsibilities	5a	Names, affiliations, and roles of protocol contributors	1
	5b	Name and contact information for the trial sponsor	1
	5c	Role of study sponsor and funders, if any, in study design; collection, management, analysis, and interpretation of data; writing of the report; and the decision to submit the report for publication, including whether they will have ultimate authority over any of these activities	12
	5d	Composition, roles, and responsibilities of the coordinating centre, steering committee, endpoint adjudication committee, data management team, and other individuals or groups overseeing the trial, if applicable (see Item 21a for data monitoring committee)	-

For peer review only

**Introduction**			
Background and rationale	6a	Description of research question and justification for undertaking the trial, including summary of relevant studies (published and unpublished) examining benefits and harms for each intervention	3,4
	6b	Explanation for choice of comparators	3,4
Objectives	7	Specific objectives or hypotheses	4
Trial design	8	Description of trial design including type of trial (eg, parallel group, crossover, factorial, single group), allocation ratio, and framework (eg, superiority, equivalence, noninferiority, exploratory)	5
**Methods: Participants, interventions, and outcomes**			
Study setting	9	Description of study settings (eg, community clinic, academic hospital) and list of countries where data will be collected. Reference to where list of study sites can be obtained	5,6
Eligibility criteria	10	Inclusion and exclusion criteria for participants. If applicable, eligibility criteria for study centres and individuals who will perform the interventions (eg, surgeons, psychotherapists)	5,6
Interventions	11a	Interventions for each group with sufficient detail to allow replication, including how and when they will be administered	8
	11b	Criteria for discontinuing or modifying allocated interventions for a given trial participant (eg, drug dose change in response to harms, participant request, or improving/worsening disease)	n.a
	11c	Strategies to improve adherence to intervention protocols, and any procedures for monitoring adherence (eg, drug tablet return, laboratory tests)	8
	11d	Relevant concomitant care and interventions that are permitted or prohibited during the trial	n.a
Outcomes	12	Primary, secondary, and other outcomes, including the specific measurement variable (eg, systolic blood pressure), analysis metric (eg, change from baseline, final value, time to event), method of aggregation (eg, median, proportion), and time point for each outcome. Explanation of the clinical relevance of chosen efficacy and harm outcomes is strongly recommended	7
Participant timeline	13	Time schedule of enrolment, interventions (including any run-ins and washouts), assessments, and visits for participants. A schematic diagram is highly recommended (see Figure)	SM1

For peer review only - http://bmjopen.bmj.com/site/about/guidelines.xhtml

Sample size	14	Estimated number of participants needed to achieve study objectives and how it was determined, including clinical and statistical assumptions supporting any sample size calculations	8,9
Recruitment	15	Strategies for achieving adequate participant enrolment to reach target sample size	6,9
**Methods: Assignment of interventions (for controlled trials)**			
Allocation:			
Sequence generation	16a	Method of generating the allocation sequence (eg, computer-generated random numbers), and list of any factors for stratification. To reduce predictability of a random sequence, details of any planned restriction (eg, blocking) should be provided in a separate document that is unavailable to those who enrol participants or assign interventions	8
Allocation concealment mechanism	16b	Mechanism of implementing the allocation sequence (eg, central telephone; sequentially numbered, opaque, sealed envelopes), describing any steps to conceal the sequence until interventions are assigned	8
Implementation	16c	Who will generate the allocation sequence, who will enrol participants, and who will assign participants to interventions	8
Blinding (masking)	17a	Who will be blinded after assignment to interventions (eg, trial participants, care providers, outcome assessors, data analysts), and how	8
	17b	If blinded, circumstances under which unblinding is permissible, and procedure for revealing a participant's allocated intervention during the trial	-
**Methods: Data collection, management, and analysis**			
Data collection methods	18a	Plans for assessment and collection of outcome, baseline, and other trial data, including any related processes to promote data quality (eg, duplicate measurements, training of assessors) and a description of study instruments (eg, questionnaires, laboratory tests) along with their reliability and validity, if known. Reference to where data collection forms can be found, if not in the protocol	-
	18b	Plans to promote participant retention and complete follow-up, including list of any outcome data to be collected for participants who discontinue or deviate from intervention protocols	n.a

Data management	19	Plans for data entry, coding, security, and storage, including any related processes to promote data quality (eg, double data entry; range checks for data values). Reference to where details of data management procedures can be found, if not in the protocol	9
Statistical methods	20a	Statistical methods for analysing primary and secondary outcomes. Reference to where other details of the statistical analysis plan can be found, if not in the protocol	9
	20b	Methods for any additional analyses (eg, subgroup and adjusted analyses)	9
	20c	Definition of analysis population relating to protocol non-adherence (eg, as randomised analysis), and any statistical methods to handle missing data (eg, multiple imputation)	9
**Methods: Monitoring**			
Data monitoring	21a	Composition of data monitoring committee (DMC); summary of its role and reporting structure; statement of whether it is independent from the sponsor and competing interests; and reference to where further details about its charter can be found, if not in the protocol. Alternatively, an explanation of why a DMC is not needed	-
	21b	Description of any interim analyses and stopping guidelines, including who will have access to these interim results and make the final decision to terminate the trial	-
Harms	22	Plans for collecting, assessing, reporting, and managing solicited and spontaneously reported adverse events and other unintended effects of trial interventions or trial conduct	-
Auditing	23	Frequency and procedures for auditing trial conduct, if any, and whether the process will be independent from investigators and the sponsor	-
**Ethics and dissemination**			
Research ethics approval	24	Plans for seeking research ethics committee/institutional review board (REC/IRB) approval	1
Protocol amendments	25	Plans for communicating important protocol modifications (eg, changes to eligibility criteria, outcomes, analyses) to relevant parties (eg, investigators, REC/IRBs, trial participants, trial registries, journals, regulators)	-

For peer review only - http://bmjopen.bmj.com/site/about/guidelines.xhtml

Consent or assent	26a	Who will obtain informed consent or assent from potential trial participants or authorised surrogates, and how (see Item 32)	6
	26b	Additional consent provisions for collection and use of participant data and biological specimens in ancillary studies, if applicable	-
Confidentiality	27	How personal information about potential and enrolled participants will be collected, shared, and maintained in order to protect confidentiality before, during, and after the trial	12
Declaration of interests	28	Financial and other competing interests for principal investigators for the overall trial and each study site	12
Access to data	29	Statement of who will have access to the final trial dataset, and disclosure of contractual agreements that limit such access for investigators	
Ancillary and post-trial care	30	Provisions, if any, for ancillary and post-trial care, and for compensation to those who suffer harm from trial participation	
Dissemination policy	31a	Plans for investigators and sponsor to communicate trial results to participants, healthcare professionals, the public, and other relevant groups (eg, via publication, reporting in results databases, or other data sharing arrangements), including any publication restrictions	13
	31b	Authorship eligibility guidelines and any intended use of professional writers	
	31c	Plans, if any, for granting public access to the full protocol, participant-level dataset, and statistical code	-
**Appendices**			
Informed consent materials	32	Model consent form and other related documentation given to participants and authorised surrogates	-
Biological specimens	33	Plans for collection, laboratory evaluation, and storage of biological specimens for genetic or molecular analysis in the current trial and for future use in ancillary studies, if applicable	-

*It is strongly recommended that this checklist be read in conjunction with the SPIRIT 2013 Explanation & Elaboration for important clarification on the items. Amendments to the protocol should be tracked and dated. The SPIRIT checklist is copyrighted by the SPIRIT Group under the Creative Commons "Attribution-NonCommercial-NoDerivs 3.0 Unported" license.

BMJ Open

Effects of a computerized diagnostic decision support tool on diagnostic quality in emergency departments – study protocol of the DDX-BRO multi-centre cluster randomized cross-over trial

Journal:	*BMJ Open*
Manuscript ID	bmjopen-2023-072649.R1
Article Type:	Protocol
Date Submitted by the Author:	02-Mar-2023

Complete List of Authors:	Marcin, Thimo; Inselspital University Hospital Bern, Department of Emergency Medicine<br>Hautz, Stefanie C.; Inselspital University Hospital Bern, Department of Emergency Medicine<br>Singh, Hardeep; Michael E DeBakey VA Medical Center, Center for Innovations in Quality, Effectiveness and Safety (IQuESt), ; Baylor College of Medicine, Department of Medicine<br>Zwaan, Laura; Erasmus Medical Center, Institute of Medical Education Research Rotterdam (iMERR)<br>Schwappach, David; University of Bern, Institute of Social and Preventive Medicine (ISPM)<br>Krummrey, Gert; Inselspital University Hospital Bern, Department of Emergency Medicine; Bern University of Applied Sciences<br>Schauber, Stefan; University of Oslo, Center for Educational Measurement and Faculty of Medicine<br>Nendaz , Mathieu; University of Geneva, Department of medicine<br>Exadaktylos, Aristomenis; Inselspital University Hospital Bern, Department of Emergency Medicine<br>Müller, Martin; Inselspital University Hospital Bern, Department of Emergency Medicine<br>Lambrigger, Cornelia; Inselspital University Hospital Bern, Department of Emergency Medicine<br>Sauter, Thomas; Inselspital University Hospital Bern, Department of Emergency Medicine<br>Lindner, Gregor; Burgerspital Solothurn, Department of Internal and Emergency Medicine<br>Bosbach, Simon; Insel Gruppe AG, Spital Tiefenau<br>Griesshammer, Ines; Insel Gruppe AG, Spital Muensingen<br>Hautz, Wolf; Inselspital University Hospital Bern, Department of Emergency Medicine

**Primary Subject Heading**:	Emergency medicine
Secondary Subject Heading:	Diagnostics, Health informatics, Health services research, Medical education and training
Keywords:	ACCIDENT & EMERGENCY MEDICINE, Health informatics <

For peer review only - http://bmjopen.bmj.com/site/about/guidelines.xhtml

	BIOTECHNOLOGY & BIOINFORMATICS, Information management < BIOTECHNOLOGY & BIOINFORMATICS, Decision Making, Quality in health care < HEALTH SERVICES ADMINISTRATION & MANAGEMENT, INTERNAL MEDICINE

Title:

Effects of a computerized diagnostic decision support tool on diagnostic quality in emergency departments – study protocol of the DDX-BRO multi-centre cluster randomized cross-over trial.

Authors:

Thimo Marcin[1], Stefanie C Hautz[1], Hardeep Singh[2], Laura Zwaan[3], David Schwappach[4], Gert Krummrey[1,5], Stefan K Schauber[6],Mathieu Nendaz[7], Aristomenis K Exadaktylos[1], Martin Müller[1], Cornelia Lambrigger[1], Thomas C Sauter[1], Gregor Lindner[1,8], Simon Bosbach[9], Ines Griesshammer[8,10], Wolf E Hautz[1]

[1] Department of Emergency Medicine, Inselspital University Hospital Bern, University of Bern, Bern, Switzerland
[2] Baylor college of Medicine, Housten, TX, USA
[3] iMED, EDASMUS, The Netherlands
[4] Institute of Social and Preventive Medicine (ISPM), University of Bern, Bern, Switzerland
[5] Bern University of Applied Sciences, Biel, Switzerland
[6] CEMO & HUS, University of Oslo, Norway
[7] Department of medicine, University of Geneva, Switzerland
[8] Bürgerspital Solothurn, Switzerland
[9] Spital Tiefenau, Inselgruppe AG, Bern, Switzerland
[10] Spital Münsingen, Inselgruppe AG, Switzerland

Correspondence: Wolf Hautz, M.D., M.M.E., Department of Emergency Medicine, Inselspital University Hospital Bern, Freiburgstrasse, 3010 Bern, Switzerland. wolf.hautz@insel.ch

Based on clinical investigation protocol, v.1.5, 10/10/2022

For peer review only

Abstract:

Introduction: Misdiagnosis occurs in about 5% of emergency department (ED) patients. Computerized diagnostic decision support systems (CDDS) suggesting differential diagnoses (DDx) to physicians aim to improve clinical reasoning and diagnostic quality. However, controlled clinical trials investigating their effectiveness and safety are absent and consequences of its use in clinical practice unknown. We aim to investigate the effect of CDDS use by ED physicians on diagnostic quality, workflow, resource consumption and patient outcomes.

Methods and analysis: This is a multicentre, outcome assessor and patient blinded, cluster-randomised, multi-period crossover superiority trial. A validated differential diagnosis generator will be implemented in four EDs and randomly allocated to a sequence of six alternating intervention and control periods. During intervention periods, the treating ED physician will be asked to consult the CDDS at least once during diagnostic workup. During control periods, physicians will not have access to the CDDS and diagnostic workup will follow usual clinical care. Key inclusion criteria will be patients' presentation to the ED with either fever, abdominal pain, syncope or a non-specific complaint as chief complaint. Primary outcome is a binary diagnostic quality risk score composed of presence of an unscheduled medical care after discharge, change in diagnosis or death during time of follow-up or an unexpected upscale in care within 24h after hospital admission. Time of follow-up is 14 days. At least 1184 patients will be included.

Secondary outcomes include length of hospital stay, diagnostics and data regarding CDDS usage, physicians' confidence calibration and diagnostic workflow.

Statistical analysis will use general linear mixed modelling methods.

Ethics and dissemination: Approved by the cantonal ethics committee of canton Berne (2022-D0002) and Swissmedic, the Swiss national regulatory authority on medical devices. Study results will be disseminated through peer-reviewed journals, open repositories and the network of investigators and the expert and patients advisory board.

ClinicalTrials.gov Identifier: NCT05346523.

For peer review only

For peer review only - http://bmjopen.bmj.com/site/about/guidelines.xhtml

Strength and limitations:

- Study addresses diagnostic error in emergency medicine, a significant patient safety topic which is under-researched
- First prospective randomized clinical trial on the effect of computerized diagnostic decision support programs in a real-world setting
- Patient relevant outcomes, including mortality and unscheduled revisits
- Multi-centre cluster randomized crossover trial with blinded outcome assessment
- Non-random selection of participating emergency rooms

Introduction

Diagnostic error in emergency medicine

Getting the right diagnosis in health care is key to provide an explanation of a patient's health problem and informs subsequent health care and treatment.[1] Diagnostic errors have been estimated to occur in about 5% of outpatient and emergency department (ED) patients, sometimes with devastating medical and economic consequences.[2,3] We have previously found clinically significant diagnostic discrepancies between ED and hospital discharge diagnosis in 12.3% of the patients, which were associated with longer hospital stay and increased mortality.[4] The causes for diagnostic error can be diverse, but one major cause is human error.[1,3,5] This includes the consideration of incomplete patient histories, failure to consider alternative diagnoses, lack of knowledge and lack of recognition of clinical findings by physicians.[5–9] An accurate diagnosis is the basis for all treatment and care and hence, improving the diagnostic process and accuracy is key to improving patient safety and outcome.

Clinical decision support systems

Within the last decades, digitalization in health care has led to a rapid evolution of various clinical decision support systems (CDS) supposed to augment clinicians in their complex decision-making processes.[10] A recent systematic literature review on electronic health record (EHR) integrated generic CDS in the ED found positive effects on various outcomes in 83% of the included articles.[11] However, they included various CDS types with heterogeneous targets and measured outcomes and studies of mixed quality. CDS that were built to support clinicians specifically in the process of diagnosis (computerized diagnostic decision support system; CDDS) have not shown such promising results as other types of CDS so far, mostly due to negative physician perception and biases, poor accuracy or poor system integration.[10]

Differential diagnosis

A key role in the diagnostic process has been attributed to differential diagnoses (DDx) as they guide physicians in considering or excluding possible diagnoses in the ongoing diagnostic process.[12] Research on diagnostic decision making found that in absence of a correct diagnostic hypothesis, physicians tried to explain evidence away that did not fit their diagnosis and that misdiagnosis occurred most when the correct diagnosis was not even considered.[13,14] Broadening the differentials has been consistently recognized as an important measure to avoid diagnostic errors.[15,16] But given the vast amount of diseases and clinical manifestations that ED physicians are confronted with, they cannot be expected to think of all possible DDx. It is likely that they either do not recall or know all of the potential DDx fitting the clinical presentation and symptoms of any given patient.

For peer review only - http://bmjopen.bmj.com/site/about/guidelines.xhtml

DDx Generators

Reminding physicians of potential DDx is the aim of DDx generators. DDx generators are built to provide physicians with a list of DDx based on clinical data input, such as patient characteristics, symptoms, findings and other factors, and were first developed in the 1970s. Since then, DDx generators have evolved in the last decades as computational methods have advanced. Nowadays, DDx generators are capable of matching input data to large electronic databases of diagnoses using varying computational methods such as Bayesian probabilities or text mining techniques,[17] and to subsequently retrieve the correct diagnosis with a acceptable accuracy. Likewise, integration in EHR systems has improved continuously and enhanced usability in clinical practice.[10]

Accuracy of DDx generators

A systematic review and meta-analysis from 2016 investigating the efficacy and utility of DDx generators found a pooled accuracy of computer suggested diagnoses of 0.7, meaning that the correct diagnosis was among the suggested DDx in 70% of the cases. However, only small improvements were seen in before and after studies, where clinicians had the opportunity to revisit their diagnoses following a DDx generator consultation.[17] However, most of the included studies were considered to be at high risk for selection, funding and publication bias and the findings of some older included studies may not be applicable to the new generation of DDx generators, given their improved accuracy and usability. Most importantly, DDx generators were applied retrospectively across the majority of the studies, reducing the external validity of the results.

Effectiveness of DDx generators and knowledge gaps

To the best of our knowledge, studies investigating the effectiveness and safety of DDx generators in 'real world' clinical setting are absent. Therefore, it is unknown to what extent the use of these CDDS actually improves the quality of medical diagnoses and consecutive health outcomes of the individual patient. In addition, research indicates that collaboration among clinicians is frequent and substantially improves diagnostic accuracy.[1,18,19] CDDS may lower ED physician's perception whether they need advice from other team members and thus reduce the likelihood of collaboration. The effect of a DDx generator on physicians' diagnostic workup of and ED collaboration in general are widely unclear. Furthermore, previous research has indicated that CDDS usage results in increased diagnostic investigations and higher costs.[20–22]

Furthermore, considering rare differential diagnoses suggested by CDDS for patients presenting with common symptoms may trigger extensive (and expensive) additional testing that would not have been conducted otherwise. Whether possible benefits justify the potentially increasing costs in our health care system remains to be determined. Currently, evidence to inform such a debate is lacking.

Aims

In this study, we aim 1) to assess the effect of the DDx generator usage on diagnostic quality and patient outcome in patients admitted to EDs, 2) to understand the influence that DDx generators have on the physicians' diagnostic workflow and the workup in the emergency departments and 3) to investigate the effect of the DDx generator on resource utilization and costs.

For peer review only - http://bmjopen.bmj.com/site/about/guidelines.xhtml

Methods

We will conduct a multi-centre, outcome assessor and patient blinded, cluster-randomized, multi-period crossover superiority trial in four Swiss EDs.

The participating EDs will be randomly allocated to two different sequences of alternating intervention and control periods of two months each (figure 1). During the intervention periods, physicians of the respective EDs will have access to the DDx generator under investigation (Isabel Pro by Isabel Healthcare Ltd) and will be asked to consult the CDDS at least once during diagnostic workup. During the control periods, the DDx generator will not be accessible to the physicians and the diagnostic process will follow usual care. No wash-out periods will be applied as no substantial cross-over effects are expected given the nature of the intervention.

The interventional trial will be accompanied by a qualitative sub-study. First, observations (Step1) will be performed. They will also be used to guide questions for interviews. If needed, a focus group (Step 2) will support this process. Subsequently, semi-structured interviews (Step 3) will be performed with physicians during an intervention phase. Observation will be repeated in a later period to assess changes in workflow, satisfaction change etc. and if necessary, interviews will be performed again (Step 4). The qualitative part observes the physicians diagnostic process, not the patient and the focus of the present manuscript is on the interventional trial.

Study sample

Patient subjects fulfilling all of the following inclusion criteria are eligible for the investigation:

- Informed Consent signed by the subject (see supplementary file 1)
- Presentation to the ED with either fever, abdominal pain, syncope or a non-specific complaint (NSC) as chief complaint. All these complaints occur frequently, can result from a large number of underlying diseases and thus provide room for diagnostic error. Furthermore, there are no universally agreed algorithms for the diagnostic workup of any of these symptoms (as there is for chest pain, for example). NSC are defined in this study as all chief complaints not included in the check list of specific complaints according to Nemec et al..[23]
- Triaged as “no acute life-threatening condition” because study inclusion would otherwise not be feasible in many cases.
- The study subject is 18 years old or older.

The presence of any one of the following exclusion criteria, patients will not be eligible for study inclusion:

- Trauma as chief complaint, because there are standardized diagnostic workups, most trauma patients receive radiographic imaging and the potential benefit of a DDx generator is questionable.
- Pregnancy (self-reported), because options for diagnostic workup are severely constrained in these patients, and presentation is mostly related to pregnancy and its complications, reducing room for error to occur and be remediated.
- Worsening of a known pre-existing condition or medical referral with a definite diagnosis, because the diagnosis is clear in this case.
- Inability to follow the informed consent and investigation procedures, e.g., due to language barriers, psychological disorder, admittance via police, detainee status.

For peer review only - http://bmjopen.bmj.com/site/about/guidelines.xhtml

- Previous enrolment into the current investigation.

Patients presenting to any of the participating EDs during the study period between June 2022 and June 2023 will be registered in the electronic health record system (EHR) and triaged by hospital staff according to clinical routine. A dedicated and trained study nurse will consecutively screen the EHR for eligible study patients. Eligible patients will be informed about the nature of the study and written informed consent will be obtained. All consenting and included study patients will be allocated automatically to the investigation group of the respective site during the respective period.

Outcomes

An advisory board composed of subject matter experts and three patient representatives have advised on outcome selection, measurement and prioritisation.

Primary outcome is a binary score indicating the presence or absence of "risk to diagnostic quality", defined as one or more of the following:

- death within 14 days after ED discharge (yes/no)
- unscheduled medical care (ED revisits, GP visits or hospitalization) within 14 days after ED discharge (yes/no)
- unexpected intensive care unit admission from ward within 24h of hospitalization (yes/no)
- diagnostic discrepancy between the ED discharge diagnosis and the latest diagnosis 14 days after ED admission (yes/no)

The primary endpoint is positive, if one or multiple of the criteria above are true, and false if none of the criteria above occur.

Secondary outcomes are

- all variables that compose the primary endpoint separately
- unscheduled ED/GP revisits after 72h and 7 days
- length of stay in the ED in hours
- length of hospital stay if hospitalized
- diagnostic tests conducted in the ED
- diagnostic tests after ED discharge
- resource consumption in the ED (costs)
- Care consumption after ED discharge
- discharge destination
- number and disease groups of DDx provided by physicians
- number of cases where the computer generated DDx list entails the diagnosis on day 14
- diagnostic error based on full chart review for a random subset of patients
- CDDS usage (timing and number of queries)

Additional outcomes are physician confidence calibration, advice seeking behaviour and collaboration assessed by physician observations, interviews and focus groups to understand how DDx generator affect diagnostic workflow in the ED and physicians' advice seeking, collaboration and confidence calibration. Table 1 provides an overview of methods used for outcome assessment. The full visit structure of the study can be found in the supplementary file 2.

For peer review only - http://bmjopen.bmj.com/site/about/guidelines.xhtml

Table 1: Primary and secondary outcomes and their method of measurement

**Primary outcome**	
**Parameter**	**Data collection**
Death within 14±4 days after ED discharge	Study nurses will review the EHR and contact the patient and GP by phone for vital status information after 14 days.
Unscheduled medical care for the same complaints within 14±4 days after ED discharge	Study nurses will contact patients by phone 14 days after discharge to collect information about follow-up medical care and diagnosis. GP or other medical care provider will be contacted when patients indicate a visit there.
IMC unit admission within 24h after ED discharge if hospitalized	Retrieved form the EHR system.
Discrepancy in ED discharge diagnosis and diagnosis 14±4 days after ED discharge	ED discharge differential diagnoses will be looked up in the ED discharge letter and registered in the study database by a study nurse. Study nurses will contact patients by phone 14 days after discharge to collect information about follow-up medical care and diagnosis. Additionally, medical records from the local hospital and the GP are reviewed. Information about the current differential diagnoses from the EHR or the GP (if available) are preferred over patient's self-reported diagnosis.<br>Two blinded physicians will independently review the ED differential diagnoses from ED discharge and at day 14 and define whether there is a discrepancy based on clinical judgement.
**Secondary outcomes**	
Number and type of diagnostic tests performed in the ED (and hospital if hospitalized)	Study nurses will retrieve the type and number of diagnostic tests performed during ED (and hospital) stay from the EHR. The following diagnostic tests (yes/no) will be recorded: Lab (Blood), Lab (Urine), Lab (Sputum), MRI, CT, Sonography, X-ray, other.<br>Additionally, the number and type of blood samples taken and consultation of specialists will be recorded.
Resource consumption during ED (and hospital if hospitalized) stay.	Resource consumption in Swiss Francs (CHF) for each patient (costs of diagnostic tests, consultations, personal and material caused) will be obtained from the hospitals administrative database to assess costs caused during the ED stay and costs caused during hospital stay if hospitalized. Costs will additional be itemized according to the hospital's cost centres.
Sick leave days	Sick leave days will be converted to Swiss francs (CHF) through the average labour productivity database of OECD
Physician confidence in ED diagnosis	Study nurses hand out an online questionnaire to the diagnosing resident physicians after patient discharge.
Discharge destination	Retrieved from EHR
ED LOS [hours]	Retrieved from EHR
Hospital LOS if hospitalized [days]	Retrieved from EHR
CDDS usage (number of queries)	Monitored by the CDDS and stored automatically in the electronic case report form (eCRF) via API.
CDDS in- and output data	Monitored by the CDDS and stored automatically in the eCRF via API.
Patient reported outcomes	Study nurses will conduct patient interviews by phone after 14 days
Diagnostic Error	Chart review will be performed for a random sample of 50 patients with a positive primary endpoint and 50 patients with a negative primary endpoint to seek for diagnostic errors using the Revised Safer Dx Instrument according to Singh et al..[24,25]
Number and type of diagnostic tests performed in the ED (and hospital if hospitalized)	Study nurses will retrieve the type and number of diagnostic tests performed during ED (and hospital) stay from the EHR. The following diagnostic tests (yes/no) will be recorded:<br>Lab (Blood), Lab (Urine), Lab (Sputum), MRI, CT, Sonography, X-ray, other.

For peer review only - http://bmjopen.bmj.com/site/about/guidelines.xhtml

	Additionally, the number of blood samples and the number of specialist consultations will be recorded.

Follow-up

Patients discharged home will be contacted and interviewed via phone by a designated and trained study nurse 14 days after the first ED visit. If patients report any medical care consumption after discharge, the corresponding medical institution (GP, hospital etc.) will be contacted to obtain the medical record. For patients who are hospitalized at follow-up, the relevant data at the time of follow-up will be obtained from the EHR system.

Blinding

Treating physicians cannot be blinded towards the patients' study allocation for obvious reasons. Patients will be blinded, i.e., they will not be informed about the current condition (intervention or control period) of the ED they present to. Also, study nurses conducting follow up interviews with patients and their general practitioners will be blinded and all raters involved in the study will be blinded (when determining whether a diagnostic discrepancy occurred and when conducting chart review to validate the measure of our primary outcome).

Randomization

Participating EDs were be randomly assigned to one of the two sequences by an independent, blinded person before start of the recruitment phase using concealed envelopes. Patients enrolled during intervention periods of the respective site will be allocated to the intervention group or to the control group if the site is in a control phase at time of enrollment.

Study intervention

The medical device software under investigation is the CDDS "Isabel Pro - the DDx Generator" from ISABEL Healthcare Ltd. The German language version will be used in the present study. A systematic review and meta-analysis found that Isabel Pro was associated with the highest accuracy of the suggested diagnoses among all investigated CDDS (pooled rate = 0.89, 95% CI = 0.83 to 0.94; I2 = 82%, p<0.001).[17] Isabel Pro is simple to use and time to enter data and obtain diagnostic suggestions takes less than a minute.[26] Additional factors supporting the choice of Isabel Pro as DDx Generator for our study is the facilitated integration in the workflow management software and the available German interface.

Isabel Pro has been developed for health professionals, i.e., the software is intended to support clinicians in broadening their differentials in the diagnostic workup. Namely, users are provided with a list of potential DDx based on patient characteristics and key symptoms entered as free text. Usage of the software itself does not require extensive training. Isabel healthcare provides training videos of 3-5 minutes length and 2-3 slides with tips for usage. We additionally provide a short training to the German interface. The most important usage instructions are also provided on the web interface of the software itself. All residents will additionally be briefed by a designated study nurse before first usage. Study nurses are monitoring adherence to the protocol and remind physicians to use the CDDS if necessary.

For peer review only - http://bmjopen.bmj.com/site/about/guidelines.xhtml

Sample Size

The sample size calculation has been performed for a multi-period cross-over cluster randomized controlled trial according to Hemming at al. (2020) using The Shiny CRT Calculator.[27] The trial is designed to have a power of 80% to detect a clinically significant between-condition-difference in the primary outcome of 5 percent points on an alpha level of 0.05.

For the primary outcome, we assumed a positive composite score in 12% of the cases in the control condition.[4] Further assumptions were a cross-sectional sampling and exchangeable correlation structure, an intra cluster correlation between 0.01 and 0.05, a coefficient of variation of cluster size of 0.5 and a 10% lost-to-follow up patients. Minimal sample size under the conditions above is 1’184 patients in total. The sample size calculation was initially performed for 4 periods, however, the trial has been extended to 6 periods during study conduct due to slow recruitment.

Statistical Analysis

Statistical analysis will be based on general linear mixed modelling (GLMM) methods using appropriate post hoc techniques (e.g for subgroup analyses).

Standard descriptive statistics, and illustrative graphing will be used throughout, along with normality testing (e.g. Shapiro-Wilk) in order to check assumptions for the appropriate use of parametric testing approaches. Transformations to normality for variables not fulfilling normality assumptions will be considered (e.g. log, Box-Cox etc.), while nonparametric testing using counterparts of ad-hoc parametric procedures will also be an option as needed (e.g. Kruskal-Wallis instead of one-way ANOVA, the latter being part of the GLM family). The R Language for Statistical Programming (R Foundation for Statistical Computing, Vienna, Austria) will be used for data analysis. A test-wise 2-sided p-value of less than 0.05 (after post-hoc and/or FDR adjustment if deemed appropriate) will be considered statistically significant.

Data will be analysed according to the intention-to-treat principle. Data from all participants with or without protocol violation including dropouts and withdrawals will be included in the main analysis. A Per-Protocol (PP) analysis will be performed as sensitivity analysis. Patients from the intervention group will be removed from the PP-analysis if no CDDS query has been documented and vice versa, patients from the control group will be removed from the analysis if physicians self-report the query of any DDx generator outside the study protocol.

For the primary outcome (presence or no presence of a positive diagnostic quality risk score), a generalized linear mixed model (GLMM) with a binomial distribution family and exchangeable correlation structure will be performed. The GLMM takes into account a random intercept for each site, resident and attending physician. Diagnosing resident and attending physicians are nested within sites. The condition (intervention and control) and the period (period 1 to 6) will be included as fixed factors under the assumption of equality of carry-over effects. Additionally, presenting chief complaint, patient’s age, sex and comorbidity index will be added as covariates.

For all secondary endpoints, summary statistics appropriate to the distribution will be tabulated by treatment group. Secondary efficacy analyses will parallel the primary analysis.

More details on statistical analyses are described in the statistical analysis plan, which will be published together with the full clinical investigational protocol and other study relevant documentation on Zenodo.org and referred to on clinicaltrials.gov and digitaldiagnosis.ch

For peer review only - http://bmjopen.bmj.com/site/about/guidelines.xhtml

Monitoring

An experienced study nurse from another clinic not involved in the study will be assigned for monitoring to check for trial documentation and source data verification. No specific audits or inspections are planned. An interim analysis for safety outcomes is planned after the end of the second period. Based on their evaluations, it will be decided by the sponsor-investigator and the site PIs if premature stopping of the clinical investigation is required.

Patient and Public Involvement

To ensure practical relevance of this project, applicability of its findings and dissemination of its results to foster implementation, we have established two study advisory boards. An expert board assembles specialists of the various areas affected by CDDS, while on the patient board, the perspective of patients and the public is represented. Both boards have separately met to advise us on the outcome measures for this project, the criteria for CDDS selection and potential strategies for dissemination. The advisory board will meet again after results of the study are available to foster dissemination and implementation. The expert board and network of the co-investigators further ensures a continuous flow of information to relevant stakeholders.

Ethics and dissemination

ClinicalTrials.gov Identifier: NCT05346523. The study was reviewed and approved by the cantonal ethics committee of canton Berne under Project-ID 2022-D0002 and Swissmedic, the Swiss national regulatory authority on medical devices. Any protocol modification will be communicated with the local PIs and study teams and approved by the ethics and Swissmedic.

Patient risk due to study participation is minimal. Informed patient consent will be obtained before patient inclusion. A patient advisory board was consulted during study design and substantially contributed to the definition of primary and secondary study outcomes.

Insights provided by this study will be disseminated to scientists, health care professionals, study participants, patient societies, industry and policymakers. Data will be submitted for publication in internationally peer-reviewed scientific journals. The privacy of each subject and confidentiality of their information shall be preserved in reports and publication of data. Minimal coded subject-level datasets and statistical codes will be published in an online repository together with the corresponding publications.

Authors contributions

WEH and TM wrote the first draft of the study protocol and of this manuscript. SCH, HS, LZ, DS, GK, SKS, MN, AKE, MM, CL, TCS, GL, SB, IG provided critical revisions and important comments on the initial drafts and approved both, the final version of the study protocol and of this manuscript. WEH obtained funding for the study. AKE, GL, SB and IG conduct the study at the emergency departments they chair.

TM - Thimo Marcin; SCH - Stefanie C Hautz; HS - Hardeep Singh; LZ - Laura Zwaan; DS - David Schwappach; GK - Gert Krummrey; SKS - Stefan K Schauber; MN - Mathieu Nendaz; AKE - Aristomenis K Exadaktylos; MM - Martin Müller; CL - Cornelia Lambrigger; TCS - Thomas C Sauter; GL - Gregor Lindner; SB - Simon Bosbach; IG - Ines Griesshammer; WEH - Wolf E Hautz

For peer review only - http://bmjopen.bmj.com/site/about/guidelines.xhtml

Funding

This work is supported by the Swiss National Science Foundation under its National Research Program NRP77, grant number 187284.

Competing interests

WEH has received research funding from the European Union, the Swiss National Science Foundation, Zoll foundation, Dräger Medical Germany, Mundipharma Research UK, MDI International Australia, Roche Diagnostics Germany, all outside the submitted work. WEH has provided paid consultancies to AO foundation Switzerland and MDI International Australia, all outside the submitted work. WEH has received financial support for a congress he chaired from EBSCO Germany, Isabel Healthcare UK, Mundipharma Medical Switzerland, VisualDx USA, all outside the submitted work.

References

1 Committee on Diagnostic Error in Health Care, Board on Health Care Services, Institute of Medicine, *et al. Improving Diagnosis in Health Care*. Washington, D.C.: : National Academies Press 2015. doi:10.17226/21794

2 Singh H, Meyer AND, Thomas EJ. The frequency of diagnostic errors in outpatient care: estimations from three large observational studies involving US adult populations. *BMJ Qual Saf* 2014;**23**:727–31. doi:10.1136/bmjqs-2013-002627

3 Newman-Toker DE, Peterson SM, Badihian S, *et al. Diagnostic Errors in the Emergency Department: A Systematic Review*. Rockville (MD): : Agency for Healthcare Research and Quality (US) 2022. http://www.ncbi.nlm.nih.gov/books/NBK588118/ (accessed 24 Jan 2023).

4 Hautz WE, Kämmer JE, Hautz SC, *et al.* Diagnostic error increases mortality and length of hospital stay in patients presenting through the emergency room. *Scand J Trauma Resusc Emerg Med* 2019;**27**:54. doi:10.1186/s13049-019-0629-z

5 Zwaan L, de Bruijne M, Wagner C, *et al.* Patient Record Review of the Incidence, Consequences, and Causes of Diagnostic Adverse Events. *ARCH INTERN MED* 2010;**170**:7.

6 Graber ML, Franklin N, Gordon R. Diagnostic Error in Internal Medicine. *Arch Intern Med* 2005;**165**:1493. doi:10.1001/archinte.165.13.1493

7 Croskerry P. The importance of cognitive errors in diagnosis and strategies to minimize them. *Acad Med* 2003;**78**:775–80.

8 Norman GR, Monteiro SD, Sherbino J, *et al.* The Causes of Errors in Clinical Reasoning: Cognitive Biases, Knowledge Deficits, and Dual Process Thinking. *Acad Med* 2017;**92**:23–30. doi:10.1097/ACM.0000000000001421

9 Zwaan L, Thijs A, Wagner C, *et al.* Relating Faults in Diagnostic Reasoning With Diagnostic Errors and Patient Harm: *Acad Med* 2012;**87**:149–56. doi:10.1097/ACM.0b013e31823f71e6

10 Sutton RT, Pincock D, Baumgart DC, *et al.* An overview of clinical decision support systems: benefits, risks, and strategies for success. *NPJ Digit Med* 2020;**3**:17. doi:10.1038/s41746-020-0221-y

For peer review only - http://bmjopen.bmj.com/site/about/guidelines.xhtml

11 Patterson BW, Pulia MS, Ravi S, *et al.* Scope and Influence of Electronic Health Record–Integrated Clinical Decision Support in the Emergency Department: A Systematic Review. *Ann Emerg Med* Published Online First: January 2019. doi:10.1016/j.annemergmed.2018.10.034

12 Jain B. The key role of differential diagnosis in diagnosis. *Diagnosis* 2017;**4**:239–40. doi:10.1515/dx-2017-0005

13 Kostopoulou O. Diagnosis of Difficult Cases in Primary Care. *J Health Serv Res Policy* 2010;**15**:71–4. doi:10.1258/jhsrp.2009.09s112

14 Kostopoulou O, Devereaux-Walsh C, Delaney BC. Missing Celiac Disease in Family Medicine: The Importance of Hypothesis Generation. *Med Decis Making* 2009;**29**:282–90. doi:10.1177/0272989X08327493

15 Singh H, Giardina TD, Meyer AND, *et al.* Types and Origins of Diagnostic Errors in Primary Care Settings. *JAMA Intern Med* 2013;**173**:418. doi:10.1001/jamainternmed.2013.2777

16 Ely JW, Kaldjian LC, D'Alessandro DM. Diagnostic Errors in Primary Care: Lessons Learned. *J Am Board Fam Med* 2012;**25**:87–97. doi:10.3122/jabfm.2012.01.110174

17 Riches N, Panagioti M, Alam R, *et al.* The Effectiveness of Electronic Differential Diagnoses (DDX) Generators: A Systematic Review and Meta-Analysis. *PLOS ONE* 2016;**11**:e0148991. doi:10.1371/journal.pone.0148991

18 Hautz WE, Kammer JE, Schauber SK, *et al.* Diagnostic performance by medical students working individually or in teams. *JAMA* 2015;**313**:303–4. doi:10.1001/jama.2014.15770

19 Kämmer JE, Hautz WE, Herzog SM, *et al.* The Potential of Collective Intelligence in Emergency Medicine: Pooling Medical Students' Independent Decisions Improves Diagnostic Performance. *Med Decis Making* 2017;**37**:715–24. doi:10.1177/0272989X17696998

20 Semigran HL, Linder JA, Gidengil C, *et al.* Evaluation of symptom checkers for self diagnosis and triage: audit study. *BMJ* 2015;:h3480. doi:10.1136/bmj.h3480

21 Kanagasingam Y, Xiao D, Vignarajan J, *et al.* Evaluation of Artificial Intelligence–Based Grading of Diabetic Retinopathy in Primary Care. *JAMA Netw Open* 2018;**1**:e182665. doi:10.1001/jamanetworkopen.2018.2665

22 Apkon M. A Randomized Outpatient Trial of a Decision-Support Information Technology Tool. *Arch Intern Med* 2005;**165**:2388. doi:10.1001/archinte.165.20.2388

23 Nemec M, Koller MT, Nickel CH, *et al.* Patients presenting to the emergency department with non-specific complaints: the Basel Non-specific Complaints (BANC) study. *Acad Emerg Med Off J Soc Acad Emerg Med* 2010;**17**:284–92. doi:10.1111/j.1553-2712.2009.00658.x

24 Singh H, Bradford A, Goeschel C. Operational measurement of diagnostic safety: state of the science. *Diagnosis* 2021;**8**:51–65. doi:10.1515/dx-2020-0045

25 Singh H, Khanna A, Spitzmueller C, *et al.* Recommendations for using the Revised Safer Dx Instrument to help measure and improve diagnostic safety. *Diagnosis* 2019;**6**:315–23. doi:10.1515/dx-2019-0012

26 Graber ML, Mathew A. Performance of a web-based clinical diagnosis support system for internists. *J Gen Intern Med* 2008;**23 Suppl 1**:37–40. doi:10.1007/s11606-007-0271-8

For peer review only

27 Hemming K, Kasza J, Hooper R, *et al.* A tutorial on sample size calculation for multiple-period cluster randomized parallel, cross-over and stepped-wedge trials using the Shiny CRT Calculator. *Int J Epidemiol* 2020;**49**:979–95. doi:10.1093/ije/dyz237

Figure Legends

Figure 1: Study Design

Word count

3286

Acknowledgements

Isabel Healthcare Ltd provided technical support to the study team in the usage of the Isabel Pro API. They further provided free exploratory access to their English as well as German API during trial setup and supported study implementation with the provision of educational material. Lastly, Isabel provided technical documentation to facilitate the regulatory evaluation of the study. Dr. Andreas Limacher of CTU Bern provided advice on sample size calculation. We are grateful to the subject matter experts and patient representatives who participated in the advisory board of the study.

For peer review only

For peer review only - http://bmjopen.bmj.com/site/about/guidelines.xhtml

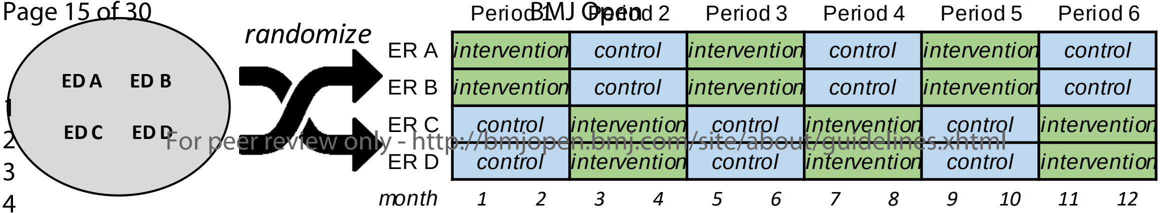

For peer review only - http://bmjopen.bmj.com/site/about/guidelines.xhtml

INSELSPITAL
UNIVERSITÄTSSPITAL BERN
HÔPITAL UNIVERSITAIRE DE BERNE

Universitäres NOTFALL ZENTRUM Erwachsene

Direktor und Chefarzt
Prof. Dr. med. Aristomenis Exadaktylos

Leitender Arzt Forschung
Prof. Dr. med. Wolf Hautz, MME

Anfrage zur Teilnahme an medizinischer Forschung:

Auswirkungen einer Diagnose-Support-Software auf die Diagnosequalität bei Notfall-Patienten

Sehr geehrte Dame, sehr geehrter Herr

Wir fragen Sie hier an, ob Sie bereit wären, an unserem Forschungsvorhaben mitzuwirken.

Ihre Teilnahme ist freiwillig. Alle Daten, die in diesem Projekt erhoben werden, unterliegen strengen Datenschutzvorschriften. Das Forschungsvorhaben wird durchgeführt von der Insel Gruppe. Bei Interesse informieren wir Sie gerne über die Ergebnisse aus dem Forschungsvorhaben.

In einem Gespräch erklären wir Ihnen die wichtigsten Punkte und beantworten Ihre Fragen. Damit Sie sich bereits jetzt ein Bild machen können, hier das Wichtigste vorweg. Im Anschluss folgen dann weitere, detaillierte Informationen.

Warum führen wir dieses Forschungsvorhaben durch?

- Kommen Patienten mit unspezifischen Symptomen (Beschwerden) in den Notfall, kommen oftmals eine Vielzahl von Diagnosen in Frage. Für die Diagnosestellung ist es wichtig, neben der hauptsächlich vermuteten Erkrankungen auch an alternative Diagnosen, sogenannte Differentialdiagnosen, zu denken.
  Dabei kann es vorkommen, dass wichtige Differentialdiagnosen vergessen gehen oder nicht in Betracht gezogen werden.

- Heutzutage gibt es Computerprogramme die Ärzte und Ärztinnen bei der Diagnosestellung unterstützen sollen. Die Fachpersonen geben relevante Daten wie Symptome in das Computerprogramm ein, welches diese dann mit einer grossen Datenbank abgleicht und passende Diagnosen vorschlägt.

- In unserem Forschungsvorhaben wollen wir herausfinden, ob sich die Diagnosequalität verbessert, wenn die behandelnden Ärzte und Ärztinnen im Notfallzentrum eine solches Computerprogramm verwenden.

Was muss ich bei einer Teilnahme tun? – Was geschieht mit mir bei einer Teilnahme?

- Ablauf: Wenn Sie sich entscheiden mitzumachen, wird der behandelnde Assistenzarzt oder die behandelnde Assistenzärztin ihre Symptome in das Computerprogramm eingeben, sofern zu dieser Zeit das Computerprogramm zur

For peer review only - http://bmjopen.bmj.com/site/about/guidelines.xhtml

INSELSPITAL
UNIVERSITÄTSSPITAL BERN
HÔPITAL UNIVERSITAIRE DE BERNE

Universitäres NOTFALL ZENTRUM Erwachsene

Verfügung steht. Das Ärzteteam entscheidet dann, ob und welche der vorgeschlagenen Differentialdiagnosen in Betracht gezogen und unter Umständen weiter abgeklärt werden sollten. Steht das Programm zu dieser Zeit nicht zur Verfügung, erfolgt die Diagnose wie üblich ohne Hilfe durch ein Computerprogramm.

- Aufwand: 14 Tage nach ihrem Notfallaufenthalt werden wir Sie telefonisch kontaktieren um mit Ihnen ein kurzes Gespräch über ihren gesundheitlichen Verlauf zu führen. Dieses Gespräch dauert ungefähr 10-20 Minuten. Ebenfalls kann ihr Hausarzt kontaktiert werden um Informationen zum gesundheitlichen Verlauf zu erhalten.

Welcher Nutzen und welches Risiko sind damit verbunden?

Nutzen

- Sie helfen mit Ihrer Teilnahme künftigen Patientinnen und Patienten.

Risiko und Belastung

- Ein direktes Risiko oder Belastung durch die Verwendung des Computerprogrammes besteht nicht. Allerdings könnte es vorkommen, dass die Ärzte und Ärztinnen zusätzliche Abklärungen vornehmen möchten um vorgeschlagene Diagnosen ausschliessen zu können. Die Verantwortung, dass keine unnötigen Abklärungen vorgenommen werden liegt nach wie vor bei den Fachpersonen und ihrer medizinischen Einschätzung.

Mit Ihrer Unterschrift am Ende des Dokuments bezeugen Sie, dass Sie freiwillig teilnehmen und dass Sie die Inhalte des gesamten Dokuments verstanden haben.

For peer review only - http://bmjopen.bmj.com/site/about/guidelines.xhtml

INSELSPITAL
UNIVERSITÄTSSPITAL BERN
HÔPITAL UNIVERSITAIRE DE BERNE

Universitäres NOTFALL ZENTRUM Erwachsene

Detaillierte Information

1. Ziel und Auswahl

Unser Forschungsvorhaben bezeichnen wir in dieser Informationsschrift als Studie. Wenn Sie an dieser Studie teilnehmen, sind Sie eine *Studienteilnehmerin* bzw. ein *Studienteilnehmer*.

Bevor ein Medizinprodukt angewendet wird, muss es bei Studienteilnehmenden wissenschaftlich untersucht werden. Wir wollen daher untersuchen, welche Auswirkungen es auf die Diagnosequalität im Notfall hat, wenn Ärzte und Ärztinnen die Medizinprodukte-Software (*Isabel – Der DDx Generator von Isabel Healthcare Ltd.*) als Unterstützung bei der Diagnosestellung verwenden.

Die Software (Computerprogramm) hat zum Ziel, Symptome und andere Patientendaten mit einer großen Datenbank an Diagnosen abzugleichen um den Fachpersonen passende Diagnosen vorzuschlagen. Die Software schlägt keine definitiven Diagnosen vor, sondern soll die Fachpersonen anregen Diagnosen in Betracht zu ziehen, welche Ihnen ansonsten entgangen wären.

Die Software schlägt gemäß durchgeführten Studien sehr oft die richtige Diagnose vor und wird bereits seit einigen Jahren in diversen Spitälern im englischsprachigem Raum verwendet. Welche Auswirkungen die Anwendung der Software auf die Diagnosestellung in Notfall-Kliniken hat wurde aber bislang noch nicht systematisch mit einer Studie untersucht.

Wir fragen Sie an, da alle Personen teilnehmen können, die mit Fieber, Bauchschmerzen, kurzzeitiger Bewusstlosigkeit oder unspezifischen Symptomen auf den Notfall kommen und nicht akut lebensbedrohlich gefährdet sind.

2. Allgemeine Informationen

- Wir wissen noch wenig über die tatsächlichen Auswirkungen, wenn Ärzte und Ärztinnen auf dem Notfall ein Computerprogramm verwenden um nach möglichen Diagnosen zu suchen.
- Wir möchten daher herausfinden, ob die Nutzung einer solchen Software die Diagnosequalität und Patientensicherheit verbessert, ob diagnostische Abklärungen zu- oder abnehmen und ob sich die Zusammenarbeit zwischen den Fachpersonen verändert.
- Die Software wird den teilnehmenden Spitälern über mehrere Zeiträume abwechslungsweise zur Verfügung gestellt (Interventionsperiode) respektive enthalten (Kontrollperiode). Wenn Sie während einem Zeitraum auf den Notfall kommen, wo die Software zur Verfügung steht, gehören Sie zur Interventionsgruppe, ansonsten zur Kontrollgruppe.
  Gehören Sie zur Interventionsgruppe werden die behandelnden Assistenzärztinnen und Assistenzärzte aufgefordert, Ihre Symptome in das Computerprogramm einzugeben um mögliche Diagnosen nachzuschlagen.
  Gehören Sie zur Kontrollgruppe haben die Assistenzärzte keinen Zugriff auf das Programm und die Diagnosestellung folgt der üblichen Behandlung. Sie erhalten keine Informationen in welche Gruppe Sie zugeteilt werden.
- Der Hersteller und die zuständige Behörde haben die Software bis zur letzten Änderung der Medizinprodukte-Regulierung nicht als Medizinprodukt klassifiziert. Aus diesem Grund hat die Software keine Medizinprodukte-Zertifizierung, wurde aber bereits auf den Markt gebracht und in Gesundheitseinrichtungen an Patienten angewendet.
- Insgesamt nehmen vier Notfallzentren aus der Region Bern und Solothurn teil. Die Dauer der Studie beträgt 8 bis 12 Monate, je nachdem wie schnell wir eine ausreichende Anzahl an Patientinnen und Patienten für diese Studie gewinnen können. Während dieser Zeit sollen insgesamt ungefähr 1'200 Patienten und Patientinnen eingeschlossen werden.
- Wir machen diese Studie so, wie es die Gesetze in der Schweiz vorschreiben. Außerdem beachten wir alle international anerkannten Richtlinien. Die zuständige Ethikkommission und Swissmedic haben die Studie geprüft und bewilligt.

For peer review only - http://bmjopen.bmj.com/site/about/guidelines.xhtml

- Eine Beschreibung dieser Studie finden Sie auch auf der Internetseite des Bundesamtes für Gesundheit unter www.kofam.ch

3. Ablauf

- Die studienbedingte Intervention (Abfrage der Diagnose-Software) erfolgt durch die behandelnden Ärzte. Sie werden davon nichts bemerken.
- Für Sie entsteht aufgrund der Studienteilnahme kein zusätzlicher Aufwand während Ihrem Notfallaufenthalt. Untersuchungen und Behandlungen erfolgen nur aufgrund medizinischer Notwendigkeit, basierend auf der Einschätzung und Verordnung ihres behandelnden Ärzteteams, im Notfallzentrum.
- Außerhalb der klinischen Routine möchten wir gerne wissen, wie es Ihnen in den 14 Tagen seit der Behandlung auf dem Notfall ergangen ist. Dazu würde Sie ein Mitarbeiter oder eine Mitarbeiterin des Studienteams der Insel Gruppe nach 14 Tagen anrufen um ein telefonisches Gespräch von rund 10-20 Minuten mit Ihnen zu führen.
- Ihre Hausärztin/Ihr Hausarzt wird über Ihre Studienteilnahme informiert. Es kann sein, dass wir Ihre Hausärztin/Ihr Hausarzt nach 14 Tagen kontaktieren um im Rahmen der Studie zusätzliche Angaben zu ihrem gesundheitlichen Verlauf zu erhalten.
- Sie werden vorzeitig aus der Studie ausgeschlossen, wenn die sichere Durchführung der Studie nicht gewährleistet werden kann und gemäß Studienteam oder Prüfperson in ihrem medizinischem Interesse liegt oder Sie selbst den Abbruch der Studie wünschen.

4. Nutzen

Es kann sein, dass die Teilnahme keinen direkten Nutzen für Sie mit sich bringt. Durch die Teilnahme an dieser Studie helfen Sie uns aber zu verstehen, ob generell die Diagnosequalität im klinischen Alltag auf dem Notfallzentrum mit einer Diagnosesoftware verbessert werden kann.

5. Freiwilligkeit und Pflichten

Sie nehmen freiwillig teil. Wenn Sie nicht an dieser Studie teilnehmen oder später Ihre Teilnahme zurückziehen wollen, müssen Sie dies nicht begründen. Ihre medizinische Behandlung/Betreuung ist unabhängig von Ihrem Entscheid gewährleistet.

Wenn Sie an dieser Studie teilnehmen, werden Sie gebeten:

- Das Studienteam beim Telefoninterview nach 14 Tagen über den Verlauf der Erkrankung zu informieren und neue Diagnosen, Symptome und Beschwerden sowie Änderungen im Befinden zu melden.

6. Risiken und Belastungen

Es kann sein, dass Ärzte und Ärztinnen durch das Computerprogramm auf mögliche Diagnosen hingewiesen werden, welche sie ansonsten nicht berücksichtigt hätten. Dies kann dazu führen, dass weiterführende Abklärungen veranlasst werden um diese Diagnosen auszuschließen und Ihr Aufenthalt auf dem Notfall verlängert wird. Diagnostische Abklärungen werden aber nur aus medizinischen Gründen durchgeführt, das heißt, wenn das Ärzteteam die weiteren Abklärungen auch als medizinisch relevant erachtet. Das Risiko für unbegründete Abklärungen ist somit gering.

7. Alternativen

Die Teilnahme an der Studie ist mit Chancen und Risiken verbunden. Wenn Sie der Studie nicht einwilligen, erhalten Sie die normale Behandlung im Notfall. Es kann aber nicht ausgeschlossen werden, dass ihre behandelnden Ärzte trotzdem ein alternatives, öffentlich zugängliches Computerprogramm innerhalb der Routinebehandlung verwenden um nach möglichen Diagnosen zu suchen.

8. Ergebnisse

Es gibt

1. individuelle Ergebnisse der Studie, die Sie direkt betreffen,
2. objektive End-Ergebnisse der gesamten Studie.

For peer review only - http://bmjopen.bmj.com/site/about/guidelines.xhtml

INSELSPITAL
UNIVERSITÄTSSPITAL BERN
HÔPITAL UNIVERSITAIRE DE BERNE

Universitäres NOTFALL ZENTRUM Erwachsene

Zu 1: Die Prüfärztin/der Prüfarzt wird Sie im Verlauf der Studie über alle für Sie persönlich wichtigen, neuen Ergebnisse und Erkenntnisse informieren. Sie werden mündlich und schriftlich informiert und können dann erneut entscheiden, ob Sie an der Studie weiter teilnehmen möchten.
Zu 2: Die objektiven End-Ergebnisse der gesamten Studie werden der fachwissenschaftlichen Öffentlichkeit zugängig gemacht und der Schweizer Aufsichtsbehörde SwissMedic und den zuständigen Ethikkomissionen zugestellt. Die Veröffentlichung, respektive Weitergabe der Ergebnisse erfolgt in verschlüsselter und/oder zusammengefasster Form, in der kein Rückschluss auf Sie als einzelnen Patienten möglich ist. Ihr Prüfärztin/Ihr Prüfarzt kann Ihnen am Ende der Studie eine Zusammenfassung der Gesamtergebnisse zukommen lassen, wenn Sie dies wünschen.

9. Vertraulichkeit von Daten

Für diese Studie werden Daten zu Ihrer Person und Gesundheit erfasst und bearbeitet, teilweise in automatisierter Form. Bei der Datenerhebung werden Ihre Daten verschlüsselt. Verschlüsselung bedeutet, dass alle Bezugsdaten, die Sie identifizieren könnten (Name, Geburtsdatum etc.), gelöscht und durch einen Code ersetzt. Personen, die keinen Zugang zu dieser Schlüssel-Liste haben, können keine Rückschlüsse auf Ihre Person ziehen. Die Schlüssel-Liste wird nach Abschluss der Studie von einer Person ausserhalb des Studienteam (Universitätsklinik für Intensivmedizin, Insel Gruppe) aufbewahrt.
Nur sehr wenige Fachpersonen werden Ihre unverschlüsselten Daten sehen und zwar nur, um Aufgaben im Rahmen der Studie zu erfüllen. Diese Personen unterliegen der Schweigepflicht. Sie als teilnehmende Person haben das Recht auf Einsicht in Ihre Daten.

Alle Vorgaben des Datenschutzes werden streng eingehalten. Es ist möglich, dass Ihre Daten in verschlüsselter Form, zum Beispiel für eine Publikation, übermittelt werden müssen und anderen Forschern zur Verfügung gestellt werden können (auch ins Ausland).
Ärztinnen und Ärzte, die für die Nachbehandlung verantwortlich sind, können kontaktiert werden, um Auskunft über Ihren Gesundheitszustand zu geben.

Diese Studie kann durch die zuständige Ethikkommission, die Arzneimittelbehörde Swissmedic oder durch den Sponsor-Investigator, der die Studie veranlasst hat, überprüft werden. Die Prüfarztin/der Prüfarzt muss dann Ihre Daten für solche Kontrollen offenlegen. Alle müssen absolute Vertraulichkeit wahren.

10. Rücktritt
Sie können jederzeit von der Studie zurücktreten. Die bis dahin erhobenen Datenwerden in diesem Fall allerdings noch verschlüsselt ausgewertet.
Im Falle eines Rücktritts bleiben Ihre Daten weiterhin verschlüsselt in den Studiendokumenten. Dies dient vorrangig Ihrer medizinischen Sicherheit. Prüfen Sie bitte, ob Sie damit einverstanden sind, bevor Sie bei der Studie mitmachen.

11. Entschädigung
Wenn Sie an dieser Studie teilnehmen, bekommen Sie dafür keine finanzielle Entschädigung. Es entstehen Ihnen oder Ihrer Krankenkasse keine Kosten durch die Teilnahme.
Die Ergebnisse dieser Studie können unter Umständen dazu beitragen, kommerzielle Produkte (z.B. Medizinprodukte-Software) zu entwickeln. Durch Ihre Studienteilnahme haben Sie kein Anrecht auf Anspruch an kommerziellen Entwicklungen (z.B. Patente).

12. Haftung
Die Insel Gruppe die die Studie veranlasst hat und für die Durchführung verantwortlich ist, haftet für Schäden, die Ihnen im Zusammenhang mit dem Prüfprodukt entstehen könnten. Die Voraussetzungen und das Vorgehen dazu sind gesetzlich geregelt.

For peer review only - http://bmjopen.bmj.com/site/about/guidelines.xhtml

Die Insel Gruppe hat daher eine Versicherung bei der *Zürich Versicherungs-Gesellschaft AG, Thurgauerstrasse 80, 8050 Zürich* abgeschlossen, um in einem möglichen Schadenfall für die Haftung aufkommen zu können.
Sollten Sie durch die Teilnahme an dieser Studie einen Schaden erleiden, so wenden Sie sich bitte an die Prüfärztin/den Prüfarzt oder an das oben erwähnte Versicherungsunternehmen.

13. Finanzierung
Die Studie wird mehrheitlich durch den Schweizerischen Nationalfonds 407740_187284 / 1 bezahlt.

14. Kontaktperson(en)
Sie dürfen jederzeit Fragen zur Studienteilnahme stellen. Auch bei Unsicherheiten oder Notfällen, die während der Studie oder danach auftreten, wenden Sie sich bitte an:

Prof. Dr. med. Wolf Hautz
Leitender Arzt Forschung
Universitäres Notfallzentrum
Inselspital Bern
Freiburgstrasse 16C, 3010 Bern
Tel.: +41 31 632 57 01
E-Mail: wolf.hautz@insel.ch

Oder an den Dienstoberarzt des Notfallzentrums
Tel.-Nr.: +41 31 632 24 02 (365 Tage, 24h besetzt)

For peer review only - http://bmjopen.bmj.com/site/about/guidelines.xhtml

INSELSPITAL
UNIVERSITÄTSSPITAL BERN
HÔPITAL UNIVERSITAIRE DE BERNE

Universitäres NOTFALL ZENTRUM Erwachsene

Einwilligungserklärung

Schriftliche Einwilligungserklärung zur Teilnahme an einer klinischen Studie
Bitte lesen Sie dieses Formular sorgfältig durch. Bitte fragen Sie, wenn Sie etwas nicht verstehen oder wissen möchten. Für die Teilnahme ist Ihre schriftliche Einwilligung notwendig.

**BASEC-Nummer (nach Einreichung):**	2022-D0002
**Titel der Studie (wissenschaftlich und Laiensprache):**	Auswirkungen einer Diagnose-Support-Software auf die Diagnosequalität bei Notfall-Patienten
**Verantwortliche Institution (Sponsor-Investigator mit Adresse):**	Prof. Dr. med. Wolf Hautz<br>Leitender Arzt Forschung<br>Universitäres Notfallzentrum<br>Inselspital Bern<br>Freiburgstrasse 16C, 3010 Bern<br>Tel.: +41 31 632 57 01
**Ort der Durchführung:**	Universitäres Notfallzentrum<br>Inselspital Bern<br>Telefon: +41 31 632 24 02
**Prüfärztin/Prüfarzt am Studienort:**<br>Name und Vorname in Druckbuchstaben:	Prof. Dr. Wolf Hautz<br>Leitender Arzt Forschung
**Teilnehmerin/Teilnehmer:**<br>Name und Vorname in Druckbuchstaben:<br>Geburtsdatum:	☐ weiblich ☐ männlich

- Ich wurde von dem oder der unterzeichnenden Prüfarzt/Prüfärztin oder der delegierten Person mündlich und schriftlich über den Zweck, den Ablauf der Studie mit der Prüfsoftware über mögliche Vor- und Nachteile sowie über eventuelle Risiken informiert.
- Ich nehme an dieser Studie freiwillig teil und akzeptiere den Inhalt der mir ausgehändigten schriftlichen Information. Ich hatte genügend Zeit, meine Entscheidung zu treffen.
- Meine Fragen im Zusammenhang mit der Teilnahme an dieser Studie sind mir beantwortet worden. Ich behalte die schriftliche Information und erhalte eine Kopie meiner schriftlichen Einwilligungserklärung.
- Ich wurde über mögliche Alternativen zur Studie, dem normalen Standardverfahren auf dem Notfall aufgeklärt.
- Ich bin einverstanden, dass meine Hausärztin/mein Hausarzt über meine Teilnahme an der Studie informiert wird.
- Im Fall einer Weiterbehandlung ausserhalb des Prüfzentrums ermächtige ich meine nachbehandelnden Ärztinnen und Ärzte, meine für die Studie relevanten Daten der Prüfärztin/dem Prüfarzt zu übermitteln.
- Ich bin einverstanden, dass die zuständigen Fachleute des Sponsor-Investigators, der zuständigen Ethikkommission und der Arzneimittelbehörde Swissmedic zu Prüf- und Kontrollzwecken in meine unverschlüsselten Daten Einsicht nehmen dürfen, jedoch unter strikter Einhaltung der Vertraulichkeit.
- Bei Ergebnissen, die direkt meine Gesundheit betreffen, werde ich informiert. Wenn ich das nicht wünsche, informiere ich meine Prüfärztin/ meinen Prüfarzt.

For peer review only - http://bmjopen.bmj.com/site/about/guidelines.xhtml

INSELSPITAL
UNIVERSITÄTSSPITAL BERN
HÔPITAL UNIVERSITAIRE DE BERNE
Universitäres NOTFALL ZENTRUM Erwachsene

- Ich weiss, dass meine gesundheitsbezogenen und persönlichen Daten nur in verschlüsselter Form zu Forschungszwecken für diese Studie weitergegeben werden können (auch ins Ausland). Der Sponsor gewährleistet, dass der Datenschutz nach Schweizer Standard eingehalten wird.
- Ich kann jederzeit und ohne Angabe von Gründen von der Studienteilnahme zu rücktreten. Meine weitere medizinische Behandlung ist unabhängig von der Studienteilnahme gewährleistet. Die bis zum Rücktritt erhobenen Daten werden noch im Rahmen der Studie ausgewertet.
- Ich bin darüber informiert, dass die Insel Gruppe eine Versicherung abgeschlossen hat, welche Schäden, die auf das Forschungsprojekt zurückzuführen sind, deckt.
- Ich bin mir bewusst, dass die in der Informationsschrift genannten Pflichten einzuhalten sind. Im Interesse meiner Gesundheit kann mich die Prüfärztin/der Prüfarzt jederzeit von der Studie ausschliessen.

Ort, Datum	Unterschrift Teilnehmerin/Teilnehmer

Bestätigung der Prüfärztin/des Prüfarztes bzw. der Prüfperson: Hiermit bestätige ich, dass ich dieser Teilnehmerin/diesem Teilnehmer Wesen, Bedeutung und Tragweite der Studie erläutert habe. Ich versichere, alle im Zusammenhang mit dieser Studie stehenden Verpflichtungen gemäss in der Schweiz geltenden Rechts zu erfüllen. Sollte ich im Verlauf der Studie von Aspekten erfahren, welche die Bereitschaft der Teilnehmerin/des Teilnehmers zur Studienteilnahme beeinflussen könnten, werde ich sie/ihn umgehend darüber informieren.

Ort, Datum	Name und Vorname der Prüfärztin/des Prüfarztes bzw. der Prüfperson in Druckbuchstaben<br><br>Unterschrift der Prüfärztin/des Prüfarztes

For peer review only

For peer review only - http://bmjopen.bmj.com/site/about/guidelines.xhtml

INVESTIGATION SCHEDULE

Investigation Periods	Screening	Consent (ICF)	Treatment, Intervention Period		Follow-up
Emergency Care in the emergency room	Admittance and Triage	Waiting Time	Medical examination and treatment		discharged or hospitalized
Time	-1 to -5h	-1 to -5h	0 to 1h	1h + LOS	14d±4d
**A)** Enrolment					
In- /Exclusion Criteria	x				
Patient information		x			
Patient consent (ICF)		x			
**B)** Intervention					
CDDS application during intervention period			x	(x)	
CDDS usage monitoring by study nurses			x		
**C) Assessments** (CR: performed within clinical routine as appropriate)					
Demographics	x				
Chief complaint	CR				
Triage assessment	CR				
Medical history			CR		
Physical examination			CR	CR	
Vital signs			CR	CR	
Laboratory tests			CR	CR	
Other diagnostic tests			CR	CR	
CDDS input / output data collection			x	(x)	
Physician questionnaire			x	(x)	
Patient telephone interviews					x
Medical record review from ER, hospital and/or GP			x	x	x
Serious Adverse Events, Adverse device effects			x	x	x
Device Deficiencies			x	x	
**D) Primary Outcome Score**					
All-cause mortality					x
Unscheduled medical care if discharged (GP, ER revisit, hospitalization)					x
Unexpected ICU admission within 24h if hospitalized					x
Current diagnosis for presenting complaint				x	x

For peer review only

**E) Secondary Outcomes**					
Number and cost of ER diagnostic tests				x	x
Time to ER diagnosis				x	
ER differential diagnoses				x	
Physician confidence in ER diagnosis				x	
Discharge destination				CR	
ER LOS				CR	
Hospital LOS if hospitalized					CR
CDDS usage (number of queries)			x	(x)	
Patient reported outcomes					x

ER, Emergency room; ICU, intensive care unit; CDDS, computerized diagnostic decision support system; GP, general practitioner; LOS, length of stay; CR, clinical routine

For peer review only

For peer review only

SPIRIT

STANDARD PROTOCOL ITEMS: RECOMMENDATIONS FOR INTERVENTIONAL TRIALS

SPIRIT 2013 Checklist: Recommended items to address in a clinical trial protocol and related documents*

Section/item	Item No	Description	Addressed on page number
**Administrative information**			
Title	1	Descriptive title identifying the study design, population, interventions, and, if applicable, trial acronym	1
Trial registration	2a	Trial identifier and registry name. If not yet registered, name of intended registry	2
	2b	All items from the World Health Organization Trial Registration Data Set	-
Protocol version	3	Date and version identifier	1
Funding	4	Sources and types of financial, material, and other support	12
Roles and responsibilities	5a	Names, affiliations, and roles of protocol contributors	1
	5b	Name and contact information for the trial sponsor	1
	5c	Role of study sponsor and funders, if any, in study design; collection, management, analysis, and interpretation of data; writing of the report; and the decision to submit the report for publication, including whether they will have ultimate authority over any of these activities	12
	5d	Composition, roles, and responsibilities of the coordinating centre, steering committee, endpoint adjudication committee, data management team, and other individuals or groups overseeing the trial, if applicable (see Item 21a for data monitoring committee)	-

For peer review only

**Introduction**			
Background and rationale	6a	Description of research question and justification for undertaking the trial, including summary of relevant studies (published and unpublished) examining benefits and harms for each intervention	3,4
	6b	Explanation for choice of comparators	3,4
Objectives	7	Specific objectives or hypotheses	4
Trial design	8	Description of trial design including type of trial (eg, parallel group, crossover, factorial, single group), allocation ratio, and framework (eg, superiority, equivalence, noninferiority, exploratory)	5
**Methods: Participants, interventions, and outcomes**			
Study setting	9	Description of study settings (eg, community clinic, academic hospital) and list of countries where data will be collected. Reference to where list of study sites can be obtained	5,6
Eligibility criteria	10	Inclusion and exclusion criteria for participants. If applicable, eligibility criteria for study centres and individuals who will perform the interventions (eg, surgeons, psychotherapists)	5,6
Interventions	11a	Interventions for each group with sufficient detail to allow replication, including how and when they will be administered	8
	11b	Criteria for discontinuing or modifying allocated interventions for a given trial participant (eg, drug dose change in response to harms, participant request, or improving/worsening disease)	n.a
	11c	Strategies to improve adherence to intervention protocols, and any procedures for monitoring adherence (eg, drug tablet return, laboratory tests)	8
	11d	Relevant concomitant care and interventions that are permitted or prohibited during the trial	n.a
Outcomes	12	Primary, secondary, and other outcomes, including the specific measurement variable (eg, systolic blood pressure), analysis metric (eg, change from baseline, final value, time to event), method of aggregation (eg, median, proportion), and time point for each outcome. Explanation of the clinical relevance of chosen efficacy and harm outcomes is strongly recommended	7
Participant timeline	13	Time schedule of enrolment, interventions (including any run-ins and washouts), assessments, and visits for participants. A schematic diagram is highly recommended (see Figure)	SM1

For peer review only - http://bmjopen.bmj.com/site/about/guidelines.xhtml

Sample size	14	Estimated number of participants needed to achieve study objectives and how it was determined, including clinical and statistical assumptions supporting any sample size calculations	8,9
Recruitment	15	Strategies for achieving adequate participant enrolment to reach target sample size	6,9
**Methods: Assignment of interventions (for controlled trials)**			
Allocation:			
Sequence generation	16a	Method of generating the allocation sequence (eg, computer-generated random numbers), and list of any factors for stratification. To reduce predictability of a random sequence, details of any planned restriction (eg, blocking) should be provided in a separate document that is unavailable to those who enrol participants or assign interventions	8
Allocation concealment mechanism	16b	Mechanism of implementing the allocation sequence (eg, central telephone; sequentially numbered, opaque, sealed envelopes), describing any steps to conceal the sequence until interventions are assigned	8
Implementation	16c	Who will generate the allocation sequence, who will enrol participants, and who will assign participants to interventions	8
Blinding (masking)	17a	Who will be blinded after assignment to interventions (eg, trial participants, care providers, outcome assessors, data analysts), and how	8
	17b	If blinded, circumstances under which unblinding is permissible, and procedure for revealing a participant's allocated intervention during the trial	8
**Methods: Data collection, management, and analysis**			
Data collection methods	18a	Plans for assessment and collection of outcome, baseline, and other trial data, including any related processes to promote data quality (eg, duplicate measurements, training of assessors) and a description of study instruments (eg, questionnaires, laboratory tests) along with their reliability and validity, if known. Reference to where data collection forms can be found, if not in the protocol	9
	18b	Plans to promote participant retention and complete follow-up, including list of any outcome data to be collected for participants who discontinue or deviate from intervention protocols	8

Data management	19	Plans for data entry, coding, security, and storage, including any related processes to promote data quality (eg, double data entry; range checks for data values). Reference to where details of data management procedures can be found, if not in the protocol	9
Statistical methods	20a	Statistical methods for analysing primary and secondary outcomes. Reference to where other details of the statistical analysis plan can be found, if not in the protocol	9
	20b	Methods for any additional analyses (eg, subgroup and adjusted analyses)	9
	20c	Definition of analysis population relating to protocol non-adherence (eg, as randomised analysis), and any statistical methods to handle missing data (eg, multiple imputation)	9
**Methods: Monitoring**			
Data monitoring	21a	Composition of data monitoring committee (DMC); summary of its role and reporting structure; statement of whether it is independent from the sponsor and competing interests; and reference to where further details about its charter can be found, if not in the protocol. Alternatively, an explanation of why a DMC is not needed	10
	21b	Description of any interim analyses and stopping guidelines, including who will have access to these interim results and make the final decision to terminate the trial	10
Harms	22	Plans for collecting, assessing, reporting, and managing solicited and spontaneously reported adverse events and other unintended effects of trial interventions or trial conduct	10
Auditing	23	Frequency and procedures for auditing trial conduct, if any, and whether the process will be independent from investigators and the sponsor	10
**Ethics and dissemination**			
Research ethics approval	24	Plans for seeking research ethics committee/institutional review board (REC/IRB) approval	1
Protocol amendments	25	Plans for communicating important protocol modifications (eg, changes to eligibility criteria, outcomes, analyses) to relevant parties (eg, investigators, REC/IRBs, trial participants, trial registries, journals, regulators)	10

For peer review only - http://bmjopen.bmj.com/site/about/guidelines.xhtml

Consent or assent	26a	Who will obtain informed consent or assent from potential trial participants or authorised surrogates, and how (see Item 32)	6
	26b	Additional consent provisions for collection and use of participant data and biological specimens in ancillary studies, if applicable	NA
Confidentiality	27	How personal information about potential and enrolled participants will be collected, shared, and maintained in order to protect confidentiality before, during, and after the trial	12
Declaration of interests	28	Financial and other competing interests for principal investigators for the overall trial and each study site	12
Access to data	29	Statement of who will have access to the final trial dataset, and disclosure of contractual agreements that limit such access for investigators	
Ancillary and post-trial care	30	Provisions, if any, for ancillary and post-trial care, and for compensation to those who suffer harm from trial participation	
Dissemination policy	31a	Plans for investigators and sponsor to communicate trial results to participants, healthcare professionals, the public, and other relevant groups (eg, via publication, reporting in results databases, or other data sharing arrangements), including any publication restrictions	13
	31b	Authorship eligibility guidelines and any intended use of professional writers	
	31c	Plans, if any, for granting public access to the full protocol, participant-level dataset, and statistical code	9
**Appendices**			
Informed consent materials	32	Model consent form and other related documentation given to participants and authorised surrogates	SM
Biological specimens	33	Plans for collection, laboratory evaluation, and storage of biological specimens for genetic or molecular analysis in the current trial and for future use in ancillary studies, if applicable	NA

*It is strongly recommended that this checklist be read in conjunction with the SPIRIT 2013 Explanation & Elaboration for important clarification on the items. Amendments to the protocol should be tracked and dated. The SPIRIT checklist is copyrighted by the SPIRIT Group under the Creative Commons "Attribution-NonCommercial-NoDerivs 3.0 Unported" license.

For peer review only - http://bmjopen.bmj.com/site/about/guidelines.xhtml
