# Supplementary material for: Effects of a computerised diagnostic decision support tool on diagnostic quality in emergency departments: study protocol of the DDx-BRO multicentre cluster randomised cross-over trial
Source: BMJ Open. 2023 Mar 28;13(3):e072649. doi: 10.1136/bmjopen-2023-072649 (PMC10069571; doi:10.1136/bmjopen-2023-072649)
Supplement: Reviewer comments [file bmjopen-2023-072649.reviewer_comments.pdf]

PEER REVIEW HISTORY

BMJ Open publishes all reviews undertaken for accepted manuscripts. Reviewers are asked to complete a checklist review form (**http://bmjopen.bmj.com/site/about/resources/checklist.pdf**) and are provided with free text boxes to elaborate on their assessment. These free text comments are reproduced below.

ARTICLE DETAILS

**TITLE (PROVISIONAL)**	Effects of a computerized diagnostic decision support tool on diagnostic quality in emergency departments – study protocol of the DDX-BRO multi-centre cluster randomized cross-over trial
**AUTHORS**	Marcin, Thimo; Hautz, Stefanie C.; Singh, Hardeep; Zwaan, Laura; Schwappach, David; Krummrey, Gert; Schauber, Stefan; Nendaz, Mathieu; Exadaktylos, Aristomenis; Müller, Martin; Lambrigger, Cornelia; Sauter, Thomas; Lindner, Gregor; Bosbach, Simon; Griesshammer, Ines; Hautz, Wolf

This article was not externally reviewed at BMJ Open. Protocols that have been independently assessed prior to submission to BMJ Open are usually fast-tracked to publication on the grounds that further substantial changes will not be possible. This independent assessment will usually be external, independent review for both a substantial grant award from a non-commercial or government funder and ethics approval.
